# The neighbourhood physical environment and active travel in older adults: a systematic review and meta-analysis

**DOI:** 10.1186/s12966-017-0471-5

**Published:** 2017-02-06

**Authors:** Ester Cerin, Andrea Nathan, Jelle van Cauwenberg, David W. Barnett, Anthony Barnett, on behalf of the Council on Environment and Physical Activity (CEPA) – Older Adults working group

**Affiliations:** 1 Institute for Health and Ageing, Australian Catholic University, Level 6, 215 Spring Street, Melbourne, VIC 3000 Australia; 2 School of Public Health, The University of Hong Kong, Hong Kong, China; 3 Baker IDI Heart and Diabetes Institute, Melbourne, Australia; 4 Department of Public Health, Ghent University, Ghent, Belgium

**Keywords:** Older adults, Active travel, Cycling, Walking, Neighbourhood, Built environment, Meta-analysis, Systematic review, Moderators

## Abstract

**Background:**

Perceived and objectively-assessed aspects of the neighbourhood physical environment have been postulated to be key contributors to regular engagement in active travel (AT) in older adults. We systematically reviewed the literature on neighbourhood physical environmental correlates of AT in older adults and applied a novel meta-analytic approach to statistically quantify the strength of evidence for environment-AT associations.

**Methods:**

Forty two quantitative studies that estimated associations of aspects of the neighbourhood built environment with AT in older adults (aged ≥ 65 years) and met selection criteria were reviewed and meta-analysed. Findings were analysed according to five AT outcomes (total walking for transport, within-neighbourhood walking for transport, combined walking and cycling for transport, cycling for transport, and all AT outcomes combined) and seven categories of the neighbourhood physical environment (residential density/urbanisation, walkability, street connectivity, access to/availability of services/destinations, pedestrian and cycling infrastructure, aesthetics and cleanliness/order, and safety and traffic).

**Results:**

Most studies examined correlates of total walking for transport. A sufficient amount of evidence of positive associations with total walking for transport was found for residential density/urbanisation, walkability, street connectivity, overall access to destinations/services, land use mix, pedestrian-friendly features and access to several types of destinations. Littering/vandalism/decay was negatively related to total walking for transport. Limited evidence was available on correlates of cycling and combined walking and cycling for transport, while sufficient evidence emerged for a positive association of within-neighbourhood walking with pedestrian-friendly features and availability of benches/sitting facilities. Correlates of all AT combined mirrored those of walking for transport. Positive associations were also observed with food outlets, business/institutional/industrial destinations, availability of street lights, easy access to building entrance and human and motorised traffic volume. Several but inconsistent individual- and environmental-level moderators of associations were identified.

**Conclusions:**

Results support strong links between the neighbourhood physical environment and older adults’ AT. Future research should focus on the identification of types and mixes of destinations that support AT in older adults and how these interact with individual characteristics and other environmental factors. Future research should also aim to clarify dose-response relationships through multi-country investigations and data-pooling from diverse geographical regions.

**Electronic supplementary material:**

The online version of this article (doi:10.1186/s12966-017-0471-5) contains supplementary material, which is available to authorized users.

## Background

Being physically active in old age is associated with numerous positive health outcomes, such as lower incidence of cognitive impairment, depression, dementia [1], coronary heart disease, some types of cancers, diabetes, stroke and hypertension [2]. The risk of all-cause mortality has been shown to be reduced by walking and cycling even after adjustment for other physical activity (PA), with the greatest impact from the first 120 min and 100 min per week for walking and cycling, respectively [3]. Active travel (AT), here defined as walking or cycling to a destination, can contribute substantiality to the accumulation of health-enhancing levels of PA in older adults. Older adults from Hong Kong, Chicago (USA) and Ghent (Belgium) reported AT accounted for 55% (169 min) of walking within the neighbourhood, 56% (159 min) of total walking and 42% (123 min) of total PA, respectively [4–6]. Changes in active commuting have been associated with corresponding changes in total PA without compensatory changes in leisure-time PA, suggesting a net benefit from engaging in AT [7, 8]. Furthermore, older people appear to experience greater overall health benefits from transport mode shifts to AT than younger people [9].

Benefits in addition to contributions to total PA are also incurred from AT. These include a quieter environment due to decreased motor vehicle noise as well as reductions in greenhouse gas emissions, air pollution, traffic congestion and transport costs [10]. Modelling has shown replacement of urban trips in private motor vehicles with AT can result in important health and economic benefits and reductions in pollutants [11–13]. Furthermore, while AT may increase exposure to air pollutants, PA benefits of AT with respect to all-cause mortality outweighed the harm caused by all but extreme levels of air pollution [14].

As opposed to planned PA (exercise), AT is typically incidental and not specifically accrued to enhance physical fitness. That is, by definition the goal of AT is to reach a destination, not to accumulate PA. AT may, therefore, not be as influenced by individual-level barriers related to participation of older adults in organised PA, such as affordability, lack of self-confidence, social awkwardness, cultural sensitivity and disinterest in PA [15].

The prevalence of PA in older adults in many countries is low [16–18]. Therefore, from a public health perspective, there is a pressing need to identify factors that can positively affect AT participation in this demographic at a population level. Perceived and objectively-assessed aspects of the neighbourhood physical environment have been postulated to be key contributors to regular engagement in AT [19]. They are defined as the objective and perceived characteristics of the physical context in which people spend their time (e.g., home, neighbourhood), including aspects of urban design (e.g., presence of sidewalks), traffic volume and speed, distance to and design of venues for PA (e.g., parks) and other destinations, crime and safety [20].

As the distance older adults travel from their home during their daily life typically decreases with age [21], the neighbourhood environment (rather than the environment around other locations) becomes increasingly important to this cohort. Also, environmental characteristics within 500 m from home appear to be more predictive of PA for older adults than those within 1–1.6 km typically used for younger age groups [22], further supporting a reduction in life space during old age. Neighbourhood characteristics facilitating engagement in AT are likely to have a large-scale, population-level effect on PA in this population. Their identification is therefore important to inform urban planning interventions.

A 2011 systematic review of associations between the physical environment and PA in older adults identified only six studies on walking for transportation and no studies on cycling for transportation [19]. Also, all these findings were from highly economically developed and low-to-medium density countries. As the number of studies has increased substantially in the last 5 years, we aimed to systematically review the literature on neighbourhood physical environmental correlates of AT in older adults. In doing so, we introduced several methodological improvements in line with the socio-ecological model of active living proposed by Sallis et al. [23], which emphasises the importance of an interactional, context-specific and domain-specific approach. For example, because environmental correlates of walking for transport appear to differ from those of cycling for transport [6, 24, 25], we examined the evidence by type of AT as well as for all types of AT combined. We also evaluated article quality and, where appropriate, reported and examined findings stratified by type of environmental measures (self-reports vs. objective) and noted moderators of environment-AT associations [26].

As noted in previous systematic reviews of environment correlates of PA [19, 20], studies in this research field typically used a variety of measures of environmental exposures and AT outcomes that precluded the conduct of a traditional meta-analysis with exact quantification of effect sizes. Consequently, previous reviews adopted an exclusively descriptive approach to the synthesis of findings relying on ‘subjective’ conclusions based on the comparison of the number of positive, zero and negative associations. This typically resulted in authors stating that there was no evidence of a relationship in cases when the frequencies of positive or negative associations were equal to or slightly lower than the frequency of nil associations. In contrast, in addition to providing a descriptive analysis of findings, this review incorporated article/study quality and sample size information into a meta-analytic approach statistically quantifying the strength of evidence for environment-AT associations. Specifically, we adapted established meta-analytic procedures to estimate the probability of observing a certain distribution of positive, negative and nil associations under a null hypothesis of no associations, therefore, providing a more robust, statistically evidence-based synthesis of findings.

## Methods

Details of the protocol for this systematic review and meta-analysis were registered in PROSPERO (Registration no. CRD42016046818 [27]).

## Search strategy, study selection and inclusion criteria

**Fig. 1:**
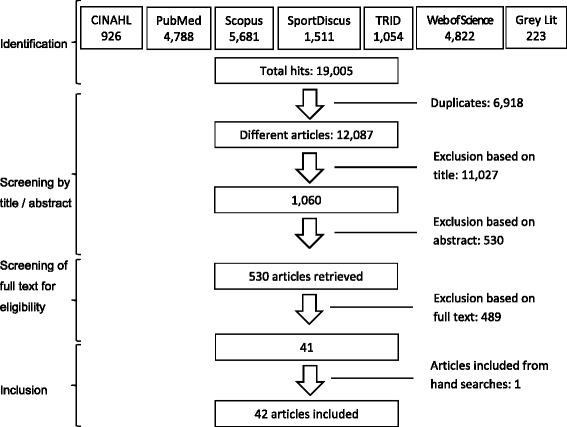
PRISMA flow chart of the systematic review. Search terms and filters used (in PubMed): (environment* OR “physical attributes” OR “physicalcharacteristics” OR “built form” OR “urban form” OR “urban design” OR neighbourhood OR neighborhood OR facilit* OR walkability OR aesthetics OR safety OR equipment OR greenness OR “park”OR “parks” OR “open space”) AND (“physical activity” OR “physical activities” OR “physically activelifestyle” OR “leisure activities” OR exercis* OR walk* OR cycle OR cycling OR commut* OR “activecommuting” OR active transport* OR “active travel”) AND (older* OR elder* OR senior* OR pensioner*) NOT (disabled OR patients OR youth OR children OR adolescent*). Filters: Language: English; Date; 01/01/2000 to 03/09/2016; Article type: books and documents, case report, government publication, journal article, meta‐analysis, observational study, review, systematic review, technical report, congress

Although this particular systematic review focuses on environmental correlates of older adults’ AT (cycling and walking for transport) only, the initial literature search aimed to provide an expanded update of Van Cauwenberg et al.’s systematic review on environmental correlates of all types of PA in older adults [19]. Van Cauwenberg et al.’s original search strategy [19] was broadened to include all types of study design, written material (grey literature) other than peer-reviewed journal articles (e.g., technical reports, proceedings, case reports and government publications) and additional key terms describing environmental exposures (i.e., physical characteristics, physical attributes, urban form, urban design, built form, greenness, park, parks and open space). A complete list of key search terms is given in Fig. 1, as they were used in PubMed. These search terms were customised for each database.

Systematic searches were performed in the following six electronic databases covering the period from January 2000 (as per Van Cauwenberg et al.’s earlier systematic review [19]) to 6th September 2016: CINAHL, PubMed, Scopus, SportDiscus, TRID and Web of Science. Additionally, a purposive sample of relevant websites was searched for grey literature (Active Living Research, SUSTRANS, National Institutes of Health, Clinical Excellence and Heart Foundation and Open Grey) and extant systematic reviews, meta-analyses and authors’ personal archives were manually screened. The searches were limited to articles in English. Three reviewers independently screened articles’ titles and abstracts for inclusion and discussed undecided cases and disagreements. Each article obtained in full text was then independently reassessed for eligibility by two reviewers and categorised according to PA outcomes into AT, leisure-time PA, total PA and/or total (not domain-specific) walking. Undecided cases and discrepancies were assessed by one or two additional reviewers and discussed with the whole team. After selecting eligible articles on AT, we re-examined the six electronic databases and Google Scholar for additional relevant output (meeting the selection criteria) by the first authors of selected articles, whereby authors’ names and their affiliations were used as search terms. We also screened the reference lists of selected articles.

Articles were included if they (a) quantitatively investigated the association between any objective or perceived attribute of the neighbourhood physical environment and a measure of AT (engagement in, frequency and/or amount of walking and/or cycling for transport); and (b) had a sample with a mean age of ≥65 years or conducted separate analyses in subsamples with a mean age of ≥65 years. We used 65 years as a cut off value because this was in line with Van Cauwenberg et al.’s review [19] and corresponded to the conventional definition of ‘older’ adults based on the qualifying state pension age in many developed countries. Articles were excluded if they (a) exclusively focused on a specific clinical population (i.e., only overweight, disabled or institutionalised participants); and/or (b) solely examined associations of AT with an ill-defined composite measure of the neighbourhood environment (e.g., studies using a study-specific, not well-established environmental index combining various poorly-correlated aspects of the built environment, such as safety and land use). Figure 1 depicts a flow chart of the systematic literature search following PRISMA guidelines [28] (see Additional file 1).

### Data extraction and validity assessment

#### Information extracted

For each included article, two reviewers extracted data, assessed study quality, verified each other’s work and resolved any discrepancies by discussion with a third reviewer and, when necessary, by contacting the authors. Data extraction was conducted using a piloted form (table) with written instructions detailing the type of information to be extracted and how to record, categorise or code it. The following information was extracted from each manuscript: (a) study name; (b) first author and year of publication; (c) sample characteristics (sample size, urban or rural location, age group, sex distribution, response rate and geographical location); (d) study design (type of study, sampling strategy for selection of study areas and participants, and neighbourhood definition); (e) list of covariates included in the analyses; (f) outcome measures (instrument, operationalisation and whether it was a validated or commonly-used measure); (g) environmental exposure measures (objective or perceived measures, attribute measured); (h) list of moderators (if any) and breakdown of sample size by categorical moderator; (i) analytical approach (type of analysis, adjustment for neighbourhood-level clustering, appropriateness of analytical approach); (j) results (associations and moderating effects); and (k) additional comments assisting the quality assessment or interpretation of the article (Additional file 2).

For analytical purposes, AT variables were categorised into: (a) *total walking for transport*; (b) *within-neighbourhood walking for transport*; (c) *cycling for transport*; and (d) *total AT* (representing measures combining walking and cycling for transport). Environmental variables were classified into categories primarily corresponding to those of the Neighborhood Environment Walkability Scale (NEWS), the most popular measure of perceived neighbourhood environmental attributes worldwide [29–31], which were complemented by several additional attributes appearing in the selected articles. Environmental variables encompassed: (a) *walkability*, denoting a composite index including information on access to services/land use mix, residential density and/or street connectivity (b) *residential density/urbanisation*; (c) *street connectivity*; (d) *access to/availability of services* with the subcategories (d.1) overall access to destinations, (d.2) land use mix – destination diversity, (d.3) shops/commercial destinations, (d.4) food outlets, (d.5) business/government/institutional/industrial destinations, (d.6) health and age-care destinations, (d.7) religious destinations, (d.8) public transport, (d.9) park/open space/recreational destinations, (d.10) entertainment and (d.11) other destinations; (e) *streetscape and pedestrian and cycling infrastructure* with the subcategories (e.1) pedestrian-friendly features, (e.2) barriers to walking/cycling, (e.3) benches/sitting facilities, (e.4) streetlights, (e.5) easy access to building entrance and (e.6) public toilets; (f) *aesthetics and cleanliness/order* with the subcategories (f.1) greenery and aesthetically pleasing scenery, (f.2) littering/vandalism/decay and (f.3) pollution (air/noise); (g) *safety and traffic* with the subcategories (g.1) traffic/pedestrian safety, (g.2) human and motorised traffic volume, and (g.3) crime/personal safety.

#### Coding and quantification of findings

Relationships between physical environmental variables and the AT outcomes were categorised as significantly positive (P), significantly negative (N) or not statistically significant (∅). Single articles were allowed to contribute with more than one finding (association) to a specific combination of environmental attribute and AT outcome if they had more than one distinct environmental variable and/or AT outcomes falling in the same categories. For example, Barnett and colleagues [4] reported two associations between land use mix – destination diversity and within-neighbourhood walking for transport - one for walking frequency, the other for volume (i.e., weekly minutes) of walking. These counted as two distinct findings (Additional file 2). Cain et al. [32] reported associations between AT and three aspects of the environment classified under the category ‘greenery and aesthetically pleasing scenery’ (trees; building aesthetic/design and positive aesthetic and social characteristics). These counted as three distinct findings (Additional file 2).

To avoid duplication of data, study findings reported by more than one article were included only if they represented original information (Additional file 2). If findings from the same study appeared in more than one article, preference was first given to those adjusted for self-selection (if available) and then to those unadjusted for other environmental variables but adjusted for socio-demographic confounders. Studies reporting multiple associations for the same combination of environmental attribute and AT outcome by different area sizes (e.g., buffers of different sizes or areas representing retirement villages vs. neighbourhoods around villages) had fractional weights assigned to each finding so that the sum of the weights across all examined associations was 1. For example, Etman et al. [33] reported nil associations between walking and aesthetics for 400 m and 1.6 km buffers around the home, while they observed positive associations for 800 m and 1.2 km buffers. In this case, each of the four findings was assigned a weight of 0.25, resulting in a summary score of 0.5 nil and 0.5 positive associations.

Studies that found significant moderators of environment-AT associations had associations reported as main effects only if the associations across all examined values of the moderator were consistent in direction and statistical significance (see King et al., [34] in Additional file 2). If this was not the case, associations at each examined value of the moderator were assigned fractional weights corresponding to the (approximate) proportion of the total sample represented by the subgroup of participants. For example, Inoue et al. [35] observed negative associations between traffic safety and walking for transport in men but not in women (i.e., sex was a moderator) (Additional file 2). As the sample consisted of 51% men, the negative association was assigned a weight of 0.51, while the nil association in women was assigned a fractional weight of 0.49. For continuous moderators, associations computed at the average value of the moderator were assigned a weight of 0.60, while those at 1 standard deviation (SD) below and above the mean were each assigned a weight of 0.20 (the total sum of the weights is 1). The logic behind this is that, under the Normal distribution, the proportions of values 1SD above and below the mean are ~ 20% (factoring some uncertainty around the value of the moderator at +1SD and -1SD). If an association was moderated by multiple factors, weights were assigned following the logic described above but in such a fashion that the sum of the weights across all examined values of all the significant moderators was 1. The weighting procedure described above was used to estimate the main effects of environmental attributes on AT, while moderators (e.g., sex and age) of associations between environmental factors and AT outcomes were examined and summarised in separate analyses.

#### Quality and sample size assessment

Article quality was assessed using nine criteria mirroring those used in other systematic reviews of environmental correlates of health-related and transportation behaviours [36, 37], and taking into account other methodological considerations relevant to this research field. These quality criteria included: (1) stratification of neighbourhoods or participants by key environmental attributes to maximise variability in exposures and outcomes [30, 38]; (2) sample shown to be representative of the population or response rate ≥60% [37]; (3) AT outcome measure shown to be valid or representing commonly-used measure [37]; (4) adjustment for socio-demographic covariates (at least age, sex, education or similar) [37]; (5) adjustment for self-selection into neighbourhoods [36]; (6) analytical approach accounted for area-level clustering (if appropriate) [39]; (7) analytical approach correctly accounted for distributional assumption of AT outcome; (8) analyses conducted and presented correctly (i.e., formal testing of moderators, if applicable; presentation of point estimates and 95% confidence intervals, standard errors and/or *p*-values); and (9) did not inappropriately categorise continuous environmental exposure [40]. Items 1–5 and 9 were each assigned a score of 1, while the three items (6–8) pertaining to statistical analyses were each assigned a score of 1/3 (i.e., 0.33). The latter was done to avoid overstating the importance of the statistical aspect of the article [by assigning a maximum of 3 rather than 1 point (0.33*3) to statistical issues] compared to other methodological issues (e.g., measurement; sample representativeness; internal validity; study design). We did not include an item assessing quality of study design in terms of strength of evidence of causality (cross-sectional, longitudinal and quasi-experimental design) because all studies included in the review were cross-sectional (Table 1). Scores on the above items were summed to yield an overall article quality score ranging from 0 to 7. Articles with a score ranging from 0 to 3.5 were deemed to be of low quality, those scoring from 3.6 to 5.9 were considered of moderate quality, and those scoring 6 to 7 were deemed high quality.

**Table 1:** Characteristics of the selected articles/studies (*N* = 42)

Characteristic	Number of articles	%
Geographical region		
Africa	1	2.4
Asia	6	14.3
Europe	9	21.4
North America	18	42.9
Oceania	5	11.9
South America	3	7.1
Geographical setting		
Urban	34	81.0
Urban, suburban and/or rural	5	11.9
Not reported	3	7.1
Study design^a^		
Cross-sectional	42	100.0
Longitudinal	0	0.0
Quasi-experimental	0	0.0
Stratification by characteristic of study area		
Area-level socio-economic status	21	50.0
Walkability aspects	22	52.4
Urbanisation	4	9.5
Demographics	3	7.1
None	12	28.6
Sample size		
≤ 100	1	2.4
101-300	7	16.7
301-500	15	35.7
501-1000	9	21.4
1001 – 2500	6	14.3
> 2500	4	9.5
Neighbourhood definition^a^		
Objective		
Administrative/census area	15	35.7
Buffer (crow-fly or road-network)		
400-500 m	9	21.4
≥ 1000 m	2	4.8
variable/not fixed	2	4.8
Perceived		
10-20 min walk from home	13	31.0
Other participant delimitation	7	16.7
Unknown	1	2.4
Studies with multiple publications		
SNQLS	6	14.3
Active Living Study	4	9.5
BEPAS Seniors	3	7.1
EpiFloripa Elderly	3	7.1
HK Elderly 1	3	7.1
Montreal’s Household Travel Survey	2	4.8
Single publication from studies with name	12	28.6
Single publication from studies with no name	9	21.4
Environmental attributes measured^a^		
Residential density/urbanisation	15	35.7
Objectively assessed	4	9.5
Perceived	11	26.2
Walkability	11	26.2
Objectively assessed	11	26.2
Perceived	0	0.0
Street connectivity	15	35.7
Objectively assessed	3	7.1
Perceived	12	28.6
Access to/availability of services and destinations^b^	33	78.6
Objectively assessed	15	35.7
Perceived	19	45.2
Pedestrian & cycling infrastructure	25	59.5
Objectively assessed	6	14.3
Perceived	19	45.2
Aesthetics and cleanliness/order	19	45.2
Objectively assessed	3	7.1
Perceived	16	38.1
Safety and traffic	24	57.1
Objectively assessed	5	11.9
Perceived	19	45.2
Active travel measures^a^ (all self-reported)		
Total walking for transport^c^	35	83.3
Continuous – frequency	5	11.9
Continuous – amount	11	26.2
Categorical – any, yes/no	10	23.8
Categorical – 60+ min/week, yes/no	5	11.9
Categorical – 150+ min/week, yes/no	1	2.4
Categorical – daily, yes/no	1	2.4
Categorical – 3 categories/levels	3	7.1
Within-neighbourhood walking for transport^c^	4	9.5
Continuous – frequency	2	4.8
Continuous – amount	4	9.5
Total cycling for transport	2	4.8
Continuous – amount	1	2.4
Categorical – daily, yes/no	1	2.4
Active travel (walking + cycling)	5	11.9
Continuous – frequency	1	2.4
Continuous – amount	3	7.1
Categorical – 3 categories/levels	1	2.4
Moderators of environment-active travel associations^a^		
Individual		
Socio-demographics	6	14.3
Psychosocial factors	1	2.4
Vehicle ownership or driving status	1	2.4
Health status/functionality	3	7.1
Environmental		
Area-level income	4	9.5
Residential density/urbanisation	2	4.8
Pedestrian infrastructure	1	2.4
Safety and traffic	2	4.8
None	25	59.5

^a^Multiple options allowed in single articles

^b^One article had both objective and perceived measures of access to/availability of services and destinations. Hence, the total number of articles is 1 unit smaller than the sum of articles with objectively assessed and perceived measures

^c^Some articles had more than one measure of walking. Hence, the total number of articles is smaller than the sum of articles with specific measures of walking

Articles were also assigned a score for sample size. The total article quality score and a sample-size score were used to compute an ‘article weight’ for the meta-analytical procedure described below. The following weights were assigned: 0.25 for a sample of ≤100 participants; 0.50 for 101–300 participants; 1.00 for 301–500 participants; 1.25 for 501–1000 participants; 1.50 for 1001–2500 participants; and 1.75 for >2500 participants. A weight of 1.00 was assigned to studies with 301–500 participants as these are ‘typical’ sample sizes in this field and, also, adequate to examine small-to-moderate associations (based on sample size calculations) [30, 41]. Non-linearly incremental weights were assigned to sample sizes because the gain in statistical power is the greatest for smaller samples and decreases as the sample size increases [42]. The sample size categories were capped at 2,501+ participants because such a sample size allows the detection of a very small effect equivalent to 1% of outcome variance explained even in presence of a substantial residual clustering effect (intra-class correlation of 0.10).

### Data synthesis

Summary of findings were given in the form of number of positive, negative and statistically non-significant associations by each combination of environmental attribute – AT outcome (e.g., shops/commercial destinations - cycling for transport) included in this review. In addition, associations were also tallied for all AT outcomes, i.e., irrespective of the type of AT, to provide a general conclusion regarding the effects of neighbourhood environmental attributes on AT. The tallying process took into account the fractional weights assigned to findings from studies examining associations by different area sizes and values of the significant moderators.

To assist the interpretation of findings and conclusions, we used a meta-analytic approach to derive conservative estimates of p-values for each combination of environmental attribute – AT outcome. This was done (a) accounting for the sample size and quality scores of the articles (see previous section); (b) accounting only for the sample size score; (c) accounting only for the quality score; and (d) accounting for neither. The last three sets of computations represented sensitivity analyses determining the impact of study quality and sample size on the meta-analytic findings. When combinations of environmental attribute – AT outcome were examined by at least three studies using objective and at least three studies using perceived measures of an environmental attribute, supplementary summaries of findings by type of environmental measure (objective vs. perceived) were conducted (*NB:* three is the typical number of studies in meta-analyses included in Cohrane’s database of systematic reviews) [43]. In line with methodological recommendations for meta-analyses [44], specific combinations of environmental attributes and AT outcomes examined less than five times were considered to be insufficiently studied.

To conduct the meta-analysis, positive associations were assigned a z-value of 1.96 (just significant at a *p*-level of 0.05), negative associations were given a z-value of −1.96 and statistically non-significant associations a z-value of 0. We did not use the exact z-values reported in the articles because the outcomes and measures were too diverse to allow the conduct of a traditional meta-analysis with accurate estimation and averaging of effect sizes. Given that, in the majority of cases, our approach underestimated the strength of associations by assigning to each significant finding the minimal z-value, the conclusions reported in this review are conservative. For each combination of environmental attribute – AT outcome variables, we computed a summary two-tailed *p*-value using Rosenthal’s approach [45], whereby a summary weighted z-value and the two-tailed probability value associated with it were obtained. As noted earlier, weights incorporated the sum of the article sample size and quality scores, which were then multiplied by the fractional weight of the z-score described above. For the three sets of sensitivity analyses, we used respectively sample size scores, quality scores and a value of 1 (i.e., meaning no weighting) as weights. The following formula was used to obtain a summary weighted z-value:

$$ Weighted\  Z = \frac{{\displaystyle \sum } weigh{t}_j{z}_j}{\sqrt{{\displaystyle \sum } weigh{t}_j^2}}, $$

where ‘j’ stands for finding ‘j’.

Following Bland’s recommendations [46], two-tailed *p*-values < 0.01 were deemed to provide strong and *p*-values < 0.001 very strong evidence of associations. A detailed example of how p-values were derived is given in the supplementary material (Additional file 3).

## Results

### Characteristics of selected articles

We screened 19,005 references and examined the full text of 530. Forty-two articles met the selection criteria for this review (Fig. 1), all reporting findings from cross-sectional studies (Table 1). Twenty-one studies were represented by a single article, while the remaining six studies had two to six articles selected for this review. Most articles reported findings from North America, followed by Europe, Asia, Oceania and South America. Africa was represented by a single article based on a small-scale, pilot study [47]. Over 80% of articles focused on older adults living in urban settings, while only five also included participants from suburban or rural areas [25, 35, 48–50].

Sample sizes ranged from 44 to 48,879 [25, 47], with eight (19.1%) articles reporting data on less than 300 participants (considered to be a small sample size in this research field) [47, 48, 50–55]. Less than 30% of articles provided sufficient evidence for the sample representativeness (Table 2). Most studies (71.4% articles; Table 2) recruited participants based on some type of stratification by environmental or socio-demographic characteristics. Fifteen articles from five studies recruited neighbourhoods stratified by both area-level socio-economic status (SES) and walkability [4, 6, 34, 51, 56]. Neighbourhoods were operationalised in a variety of ways, the most frequent of which were administrative/census areas (35.7%), 400–500 m buffers around participants’ residential addresses (21.4%) and a participant-perceived area within a 10–20 min walk from home (31.0%).

**Table 2:** Summary of article quality assessment (*N* = 42)

Quality-assessment item [score]	Number of studies	%
1. Study areas or participant recruitment stratified by key environmental attributes [1]	30	71.4
2. Response rate ≥60% or sample representative of the population [1]	12	28.6
3. Active travel measures (outcomes) valid, or well-established in the field [1]	32	76.2
4. Analyses adjusted for key socio-demographic covariates (at least age, sex and education considered) [1]	38	90.5
5. Analyses adjusted for self-selection [1]	4	9.5
6. Analytical approach – adjustment for clustering (if needed) [1/3]	30	71.4
7. Analytical approach – accounting for distributional assumptions [1/3]	36	85.7
8. Analytical approach – analyses conducted and presented correctly [1/3]	35	83.3
9. Did not (inappropriately) categorise continuous environmental exposures [1]	33	78.6
Total quality score [theoretical range: 0–7], mean (SD)	4.3	1.3

AT was gauged using valid self-report measures in 76.2% of the cases (Table 2). Most articles (83.3%) presented findings on environmental correlates of total walking for transport operationalised as a categorical, discrete or continuous outcome using a wide variety of questionnaires and different criteria of categorization/dichotomization. Only four articles, all from Hong Kong [4, 56–58], examined within-neighbourhood walking and two Belgian articles studied cycling for transport [6, 25]. Five studies across four continents used a measure of AT combining walking and cycling [32, 34, 47, 53, 59].

Access to/availability of services and destinations was the environmental category most frequently examined (78.6% of articles), followed by pedestrian/cycling infrastructure and streetscape (59.5%), and safety and traffic (57.1%). Neighbourhood environmental attributes were more frequently gauged via self-reports than objective measures, with the exception of walkability which was exclusively assessed using objective Geographic Information Systems (GIS) data. Fifteen out of 23 articles reporting on self-report measures of the neighbourhood environment used one of the versions of the NEWS [29–31]. A variety of individual and environmental moderators of environmental correlates of AT were examined in 17 (40.5%) articles (Table 1). The most frequently studied moderators were area-level income and age.

While most studies adjusted the analyses for key socio-demographic covariates, only a few considered residential self-selection (Table 2) [41, 54, 60, 61]. The adopted analytical approaches were adequate in the majority of cases, with non-adjustment for clustering and inappropriate categorisation of continuous environmental exposures being the most common problems. Detailed characteristics of each article, including quality assessment, are reported in Additional files 1 and 4.

Below, we present a detailed overview of the results separately for each AT outcome. Table 3 summarises the overall associations of environmental correlates with AT outcomes, while Table 4 summarises associations stratified by type (objective or perceived) of environmental measure for combinations of environmental attributes and AT outcomes with at least three findings per type of environmental measure. Finally, Table 5 reports moderators of environmental correlates of AT.

**Table 3:** Summary table of associations of neighbourhood physical environmental correlates of active travel in older adults

Environmental attributes	Total walking					Within-neighbourhood walking					Walking + cycling					Cycling					All active travel				
	P	∅	N	p_a_	D_a_	P	∅	N	p_a_	D_a_	P	∅	N	p_a_	D_a_	P	∅	N	p_a_	D_a_	P	∅	N	p_a_	D_a_
Residential density/urbanisation	**7**	**6**	**0**	**<.001**	**P**	1	3	0	.319	∅	1	1	0	.089	∅	0	0	1	.050	N	**9**	**10**	**1**	**.002**	**P**
Walkability	**8**	**1**	**0**	**<.001**	**P**	-	-	-	-	-	1	0	0	.050	P	0	1	0	1.00	∅	**9**	**2**	**0**	**<.001**	**P**
Street connectivity	**5**	**10**	**0**	**.014**	**P**	**2**	**2**	**0**	**.046**	**P**	0	1	0	1.00	∅	-	-	-	-	-	**7**	**13**	**0**	**.002**	**P**
Access to/availability of services/destinations																									
Overall access to destinations/services	**6.5**	**7.5**	**0**	**<.001**	**P**	**4**	**0**	**0**	**<.001**	**P**	0	1	0	1.00	∅	0	1	0	1.00	∅	**10.5**	**9.5**	**0**	**<.001**	**P**
Land use mix – destination diversity	**10.5**	**6.5**	**0**	**<.001**	**P**	**2.66**	**1.34**	**0**	**.003**	**P**	1	1	0	.310	∅	-	-	-	-	-	**14.16**	**8.84**	**0**	**<.001**	**P**
Shops/commercial	**5.33**	**3.67**	**0**	**<.001**	**P**	0.8	1.2	0	.156	∅	1	0	0	.050	P	1	0	0	.050	P	**8.13**	**4.87**	**0**	**<.001**	**P**
Food outlets	1	5	0	.542	∅	**1.8**	**2.2**	**0**	**.050**	**P**	1	0	0	.050	P	-	-	-	-	-	**3.8**	**7.2**	**0**	**.027**	**P**
Business/government/institutional/industrial	4	5	1	.112	∅	0	2	0	1.00	∅	2	0	0	.006	P	-	-	-	-	-	**6**	**7**	**1**	**.018**	**P**
Health and aged-care	2	3	1	.451	∅	1	1	0	.166	∅	-	-	-	-	-	-	-	-	-	-	3	4	1	.166	∅
Religious	0	2	0	1.00	∅	1	1	0	.166	∅	-	-	-	-	-	-	-	-	-	-	1	3	0	.327	∅
Public transport	**8.2**	**1.8**	**0**	**<.001**	**P**	0	5	0	1.00	∅	0	1	0	1.00	∅	1	0	0	.050	P	**9.2**	**7.8**	**0**	**<.001**	**P**
Parks/open space/recreation	**6.93**	**10.07**	**0**	**.001**	**P**	**2.8**	**1.2**	**0**	**.002**	**P**	1	3	0	.287	∅	-	-	-	-	-	**10.73**	**14.27**	**0**	**<.001**	**P**
Entertainment	0	5	0	1.00	∅	0.8	1.2	0	.191	∅	0	1	0	1.00	∅	-	-	-	-	-	0.8	7.2	0	.553	∅
Other	0	2.14	0.86	.310	∅	-	-	-	-	-	0	1	0	1.00	∅	-	-	-	-	-	0	3.14	0.86	.390	∅
Pedestrian & cycling infrastructure																									
Pedestrian-friendly features	**6.76**	**18.24**	**1**	**.024**	**P**	**5.22**	**6.78**	**0**	**.003**	**P**	1.45	2.55	0	.194	∅	-	-	-	-	-	**13.43**	**27.57**	**1**	**<.001**	**P**
Barriers to walking/cycling	1	10	0	.535	∅	1	3	2	.397	∅	0	2	1	.295	∅	-	-	-	-	-	2	15	3	.664	∅
Benches/sitting facilities	**2**	**2**	**0**	**.048**	**P**	**2.33**	**2.67**	**0**	**.033**	**P**	-	-	-	-	-	0.18	0.82	0	.674	∅	**4.51**	**5.49**	**0**	**.004**	**P**
Street lights	1	3	0	.290	∅	1	0	0	.050	P	1	0	0	.050	P	0.22	0.78	0	.595	∅	**3.22**	**3.78**	**0**	**.013**	**P**
Easy access to building entrance	2.56	0.44	0	.002	P	**2.49**	**1.51**	**0**	**.010**	**P**	-	-	-	-	-	-	-	-	-	-	**5.05**	**1.95**	**0**	**<.001**	**P**
Public toilets	0	2	0	1.00	∅	0	1	0	1.00	∅	-	-	-	-	-	0	1	0	1.00	∅	0	4	0	1.00	∅
Aesthetics and cleanliness/order																									
Greenery and aesthetically pleasing scenery	3.01	11.99	0	.189	∅	0.84	2.16	0	.239	∅	0	5	0	1.00	∅	0	1	0	1.00	∅	3.85	20.15	0	.160	∅
Littering/vandalism/decay	**0.72**	**2.28**	**3**	**.050**	**N**	0	3	0	1.00	∅	1	0	1	.802	∅	0.07	0.93	0	.883	∅	1.79	6.21	4	.191	∅
Pollution (air, noise)	1	2	0	.293	∅	-	-	-	-	-	0	2	0	1.00	∅	0.07	0.84	0.09	.963	∅	1.07	4.84	0.09	.457	∅
Safety and traffic																									
Traffic/pedestrian safety	3	19.49	1.51	.484	∅	0	8.78	2.22	.189	∅	3	2	1	.056	∅	0	1	1	.166	∅	6	31.27	5.73	.888	∅
Human or motorised traffic volume	2	1	0	.024	P	2	2	0	.054	∅	-	-	-	-	-	-	-	-	-	-	**4**	**3**	**0**	**.004**	**P**
Crime/personal safety	2	15.86	3.14	.667	∅	3	4	3	1.00	∅	0	3	0	1.00	∅	-	-	-	-	-	5	22.86	6.14	.755	∅

Notes. P = positive association; ∅ = nil association; N = negative association; *p* = *p* value; D = direction of associations supported by the data; subscript ‘a’ = fully adjusted (for sample size and study quality). In bold: statistically significant evidence of associations when 5+ findings reported on specific combinations of environment – active travel variables)

**Table 4:** Summary table of associations of neighbourhood physical environmental correlates with active travel in older adults by type of environmental measure (objective vs. perceived)

Environmental attributes	Total walking					All active travel				
	P	∅	N	p_a_	D_a_	P	∅	N	p_a_	D_a_
Residential density/urbanisation	7	6	0	<.001	P	9	10	1	.002	P
Objective	2	1	0	.031	P	**3**	**1**	**1**	**.145**	**∅**
Perceived	5	5	0	.004	P	**6**	**9**	**0**	**.006**	**P**
Street connectivity	5	10	0	.014	P	7	13	0	.002	P
Objective	**2**	**2**	**0**	**.041**	**P**	2	2	0	.041	P
Perceived	**3**	**8**	**0**	**.113**	**∅**	5	11	0	.014	P
Access to/availability of services/destinations										
Land use mix – destination diversity	10.5	6.5	0	<.001	P	14.16	8.84	0	<.001	P
Objective	**2.5**	**3.5**	**0**	**.051**	**∅**	**2.5**	**3.5**	**0**	**.051**	**∅**
Perceived	**8**	**3**	**0**	**<.001**	**P**	**11.66**	**5.34**	**0**	**<.001**	**P**
Public transport	8.2	1.8	0	<.001	P	9.2	7.8	0	<.001	P
Objective	4.2	0.8	0	<.001	P	4.2	2.8	0	.002	P
Perceived	4	1	0	<.001	P	5	5	0	.002	P
Parks/open space/recreation	6.93	10.07	0	.001	P	10.73	14.27	0	<.001	P
Objective	**2**	**8**	**0**	**.229**	**∅**	**2.8**	**11.2**	**0**	**.130**	**∅**
Perceived	**4.93**	**2.07**	**0**	**<.001**	**P**	**7.93**	**3.07**	**0**	**<.001**	**P**
Pedestrian & cycling infrastructure										
Pedestrian-friendly features	6.76	18.24	1	.024	P	13.43	27.57	1	<.001	P
Objective	2.25	5.75	0	.090	∅	3.25	7.75	0	.044	P
Perceived	4.51	12.49	1	.114	∅	10.18	19.82	0	<.001	P
Aesthetics and cleanliness/order										
Greenery and aesthetically pleasing scenery	3.01	11.99	0	.189	∅	3.85	20.15	0	.160	∅
Objective	0.5	2.50	0	.569	∅	0.5	5.50	0	.723	∅
Perceived	2.51	9.49	0	.237	∅	3.35	14.65	0	.154	∅
Safety and traffic										
Traffic/pedestrian safety	3	19.49	1.51	.484	∅	6	31.27	5.73	.888	∅
Objective	1	3	1	1.00	∅	**4**	**4**	**1**	**.043**	**P**
Perceived	2	16.49	0.51	.432	∅	**2**	**27.27**	**4.73**	**.398**	**∅**

Notes. P = positive association; ∅ = nil association; N = negative association; *p* = *p* value; D = direction of associations supported by the data; subscript ‘a’ = fully adjusted (for sample size and study quality). Differences in associations between objective and perceived measures of environmental attributes are bolded

**Table 5:** Overview of moderators of neighbourhood physical environmental correlates of active travel in older adults

Moderators	Environmental attribute (E) – AT outcome (AT)	Findings
Individual: socio-demographics (self-reported)		
Age (Barnes et al., in press) [62]	E: (1) Walkability; (2) Public transport AT: (1) Total walking	• No significant moderating effects.
Age (Shigematsu et al., 2009) [63]	E: (1) Residential density; (2) Access to destinations/services; (3) Land use mix – destination diversity; (4) Street connectivity; (5) Pedestrian-friendly features; (6) Greenery and aesthetically pleasing scenery; (7) Traffic/pedestrian safety; (8) Public transport; (9) Crime/personal safety; (10) Parks/open space/recreation destinations AT: (1) Total walking	• Positive associations with (10) Parks/open space/recreation (park near home) only in 75+ year olds.
Sex (Inoue et al., 2011) [35]	E: (1) Residential density; (2) Shops/commercial destinations; (3) Public transport; (4) Pedestrian-friendly features; (5) Traffic/pedestrian safety; (6) Crime/personal safety; (7) Park/open space/recreation; (8) Greenery and aesthetically pleasing scenery AT: (1) Total walking	• Positive associations with (2) Shops/commercial destinations and (7) Park/open space/recreation destinations only in women. • Positive associations with (4) Pedestrian-friendly features and (8) Greenery and aesthetically pleasing scenery only in men. • Negative associations with (5) Traffic/pedestrian safety and (6) Crime/personal safety only in men.
Age Sex Education (Cerin et al., 2014) [58]	E: (1) Residential density; (2) Access to destinations/services; (3) Land use mix – destination diversity; (4) Street connectivity; (5) Pedestrian-friendly features; (6) Traffic/pedestrian safety; (7) Public transport; (8) Crime/personal safety; (9) Barriers to walking/cycling; (10) Easy access to building entrance; (11) Human or motorised traffic volume; (12) Benches/sitting facilities AT: (1) Total walking; (2) Within-neighbourhood walking	• Positive associations of (3) Land use mix – destination diversity and (12) Benches/sitting facilities with (2) Within-neighbourhood walking only in 75+ year-olds. • Negative associations of (8) Crime/personal safety and (2) Within-neighbourhood walking only in women.
Age Sex (Van Cauwenberg et al., 2012) [25]	E: (1) Access to destinations/services; (2) Shops/commercial destinations; (3) Public transport; (4) Public toilets; (5) Benches/sitting facilities; (6) Traffic/pedestrian safety; (7) Pedestrian-friendly features; (8) Crime/personal safety; (9) Street lights; (10) Littering/vandalism/decay; (11) Pollution; (12) Greenery and aesthetically pleasing scenery AT: (1) Total walking; (2) Cycling	• Positive associations with (10) Littering/vandalism/decay and (1) Total walking in all but 75+ year-old women. • Complex Age by Sex interaction on (11) Pollution (1) Total walking associations. All associations positive. • Significant Age by Sex interaction on (4) Public toilets, (6) Traffic/pedestrian safety and (2) Cycling associations. However, no significant associations in subgroups (public toilets; presence of crossings) or all associations negative (traffic safety). • Positive associations of (9) Street lights with (2) Cycling only in <75-year old women. • See also interactions of Urbanism by Age or Sex below.
Living arrangements (Tsai et al., 2013) [64]	E: (1) Traffic/pedestrian safety; (2) Barriers to walking/cycling; (3) Easy access to building entrance; (4) Land use mix – destination diversity AT: (1) Total walking	• Positive association with (3) Easy access to building entrance only in those living alone.
Individual: psychosocial factors (perceived)		
Social support for physical activity Self-efficacy for physical activity Perceived barriers to physical activity (Carlson et al., 2012) [65]	E: (1) Walkability; (2) Parks/open space/recreation destinations AT: (1) Total walking	• Stronger associations with (1) Walkability in those with higher social support and self-efficacy, and lower perceived barriers to physical activity.
Individual: vehicle ownership/driving status (self-reported)		
Driving status (Ding et al., 2014) [22]	E: (1) Residential density; (2) Access to destinations/services; (3) Land use mix – destination diversity; (4) Street connectivity; (5) Pedestrian-friendly features; (6) Greenery and aesthetically pleasing scenery; (7) Traffic/pedestrian safety; (8) Public transport; (9) Crime/personal safety; (10) Walkability; (11) Parks/open space/recreation destinations AT: (1) Total walking	• No significant moderating effects.
Individual: health status/functionality		
Frailty (self-reported) (Etman et al., 2014) [33]	E: (1) Greenery and aesthetically pleasing scenery; (2) Pedestrian-friendly features; (3) Traffic/pedestrian safety; (4) Crime/personal safety; (5) Land use mix – destination diversity AT: (1) Total walking	• No significant moderating effects.
Chronic conditions (genitourinary, vision impairment, hearing impairment, musculoskeletal) (objective)(Barnett et al., 2016) [4]	E: (1) Residential density; (2) Access to destinations/services; (3) Land use mix – destination diversity; (4) Street connectivity; (5) Pedestrian-friendly features; (6) Greenery and aesthetically pleasing scenery; (7) Traffic/pedestrian safety; (8) Public transport; (9) Crime/personal safety; (10) Barriers to walking/cycling; (11) Parks/open space/recreation destinations; (12) Easy access to building entrance; (13) Human or motorised traffic volume; (14) Littering/vandalism/decay; (15) Benches/sitting facilities AT: (1) Within-neighbourhood walking	• Stronger positive associations with (2) Access to destination/services, (5) Pedestrian-friendly features and (7) Traffic/pedestrian safety in those with than without genitourinary diseases. • Stronger positive associations with (6) Greenery and aesthetically pleasing scenery in those without vision impairment. • Stronger positive associations with (12) Easy access to building entrance in those with than without musculoskeletal diseases.
Mobility impairment (self-reported) (King et al., 2011) [34]	E: (1) Walkability AT: (1) Walking + cycling	• Stronger positive associations in least mobility impaired.
Environmental: area-level income (objective)		
Area-level household income (King et al. 2011) [34]	E: (1) Walkability AT: (1) Walking + cycling	• No significant moderating effects.
Area-level socio-economic status (SES) (Kolbe-Alexander et al., 2015) [47]	E: (1) Residential density; (2) Access to destinations/services; (3) Land use mix – destination diversity; (4) Street connectivity; (5) Pedestrian-friendly features; (6) Greenery and aesthetically pleasing scenery; (7) Traffic/pedestrian safety; (8) Crime/personal safety AT: (1) Walking + cycling	• Positive associations with (5) Pedestrian-friendly features only in high-SES areas.
Area-level household income (Van Holle et al. 2014) [6]	E: (1) Walkability AT: (1) Total walking; (2) Cycling	• No significant moderating effects.
Area-level household income (Van Cauwenberg et al. 2016) [61]	E: (1) Walkability AT: (1) Total walking	• No significant moderating effects.
Environmental: residential density/urbanisation (objective)		
Urbanisation (Maisel, 2016) [48]	E: (1) Residential density; (2) Access to destinations/services; (3) Land use mix – destination diversity; (4) Street connectivity; (5) Pedestrian-friendly features; (6) Greenery and aesthetically pleasing scenery; (7) Traffic/pedestrian safety; (8) Crime/personal safety AT: (1) Total walking	• No significant moderating effects.
Urbanisation (Van Cauwenberg et al., 2012) [25]	E: (1) Access to destinations/services; (2) Shops/commercial destinations; (3) Public transport; (4) Public toilets; (5) Benches/sitting facilities; (6) Traffic/pedestrian safety; (7) Pedestrian-friendly features; (8) Crime/personal safety; (9) Street lights; (10) Littering/vandalism/decay; (11) Pollution; (12) Greenery and aesthetically pleasing scenery AT: (1) Total walking; (2) Cycling	• Positive associations of (2) Shops/commercial destinations with (1) Total walking in all participants but <75 year olds living in rural areas. • Significant Urbanisation by Sex interaction on (4) Public toilets and (2) Cycling associations. However, no significant associations in subgroups. • Significant positive associations between (5) Benches/sitting facilities and (2) Cycling only in rural women. • Significant positive associations between (10) Littering/vandalism/decay and (2) Cycling only in urban <75 year-old men. • Significant negative associations between (11) Pollution (noise) and (2) Cycling only in rural <75 year-old women.
Environmental: pedestrian infrastructure and streetscape (objective)		
Sloping streets Public facilities Good path conditions Path obstructions Street lights (Cerin et al., 2013) [57]	E: (1) Health and aged-care; (2) Religious destinations; (3) Public transport; (4) Parks/open space/recreation destinations; (5) Business/government/institutional/industrial); (6) Entertainment; (7) Shops/commercial; (8) Food outlets AT: (1) Total walking; (2) Within-neighbourhood walking	• Stronger positive associations between (7) Shops/commercial destinations and (2) within-neighbourhood walking in areas with more path obstructions and fewer sloping streets. • Stronger positive associations between (7) Food outlets (shops and grocery stores) and (2) within-neighbourhood walking in areas with fewer path obstructions and no sloping streets.
Environmental: safety and traffic		
Stray animals (objective) Signs of crime/disorder (objective) Pedestrian safety (objective) (Cerin et al., 2013) [57]	E: (1) Health and aged-care; (2) Religious destinations; (3) Public transport; (4) Parks/open space/recreation destinations; (5) Business/government/institutional/industrial); (6) Entertainment; (7) Shops/commercial; (8) Food outlets AT: (1) Total walking; (2) Within-neighbourhood walking	• Stronger positive associations between (3) Public transport and (1) Total walking; and (4) Parks/open space/recreation destinations and (2) Within-neighbourhood walking in areas with fewer stray animals. • Stronger positive associations of (4) Parks/open space/recreation destinations and (6) Entertainment and (2) Within-neighbourhood walking in areas with fewer signs of crime/disorder.
Traffic safety (perceived) Pedestrian safety (perceived) Crime safety (perceived) (Bracy et al., 2014) [66]	E: (1) Walkability AT: (1) Total walking	• No significant moderating effects.

### Neighbourhood environmental correlates of total walking for transport

After accounting for sample size and article quality, strong evidence (*p* < .01) of positive associations with total walking for transport was found for residential density/urbanisation, walkability, easy access to building entrance and several measures of access to/availability of services/destinations. The latter included overall access to destinations/services, land use mix – destination diversity, shops/commercial destinations, parks/open spaces/recreational destinations and public transport. Weaker evidence (*p* = 0.014 to 0.050) of positive associations was found for aspects of pedestrian infrastructure (pedestrian-friendly features; benches/sitting facilities), street connectivity, traffic volume and lack of littering/vandalism/decay. No sufficient evidence of associations was found for the remaining 13 environmental attributes, eight of which were examined five to 24 times. Among the attributes sufficiently examined (i.e., with five or more findings), the most consistent patterns of positive associations were observed for walkability, public transport, land use mix – destination diversity, shops commercial destinations, while the most consistent patterns of nil associations were found for barriers to walking/cycling, entertainment and food outlets. With the exception of two environmental characteristics (benches/sitting facilities and littering/vandalism/decay), the above conclusions held true in analyses unadjusted for sample size, article quality or both (Additional file 5).

The evidence of a positive association between street connectivity and total walking for transport was stronger for objective than perceived measures of street connectivity (Table 4). The opposite was true for land use mix – destination diversity and parks/open space/recreation. No differences in patterns of associations with total walking for transport were observed between objective and perceived measures of residential density/urbanisation, public transport, pedestrian-friendly features, greenery and aesthetically pleasing scenery and traffic/pedestrian safety.

#### Moderators of associations

Age was investigated as a moderator of associations between environmental attributes and total walking for transport in four different studies (Table 5) [25, 58, 62, 63]. Only a few significant moderating effects were observed, with stronger positive associations of parks with total walking for transport being reported in older participants (75+ year olds) in the USA [63]. In contrast, in a Belgian study, younger participants (<75 year olds) showed stronger associations with littering/vandalism/decay and shops/commercial destinations [25]. Sex was examined as a moderator in Japanese [35], Hong Kong [58] and Belgian samples [25]. While sex did not moderate the effects of the neighbourhood environment on total walking in Hong Kong older adults [58], weaker associations were found with a measure of littering/vandalism/decay among older women than other socio-demographic subgroups in Belgium [25]. Access to shops and exercise facilities were found to be more strongly positively associated with total walking for transport in Japanese women than men, while men showed stronger associations with aspects related to safety, aesthetics and pedestrian infrastructure [35]. Other socio-demographic moderators (education and living arrangement) were only examined in single studies [58, 64], as were psychosocial factors [65], driving status [22], and frailty [33].

Two articles from the same Belgian study estimated the moderating effect of area-level income on walkability and total walking for transport and found none [6, 61]. Two articles also examined level of urbanisation as a moderator of a wide range of perceived neighbourhood attributes and total walking for transport. No evidence was found in the USA [48] and very limited evidence in Belgium [25], where stronger associations between shops and walking were detected in more urban areas (Table 5). Using environmental measures derived from detailed neighbourhood audits, a study conducted in Hong Kong examined aspects of pedestrian infrastructure and safety as moderators of associations between access/availability of destinations and total walking for transport [57]. Only the presence of stray animals was found to modify the association between prevalence of public transport points and walking, whereby residents of neighbourhood with no or few stray animals showed stronger positive associations than their counterparts. No significant moderating effects of aspects of perceived safety on the associations between walkability and total walking for transport were found in a USA sample [66].

### Neighbourhood environmental correlates of within-neighbourhood walking for transport

Only four articles from two Hong Kong studies examined neighbourhood environmental correlates of within-neighbourhood walking [4, 56–58]. Strong evidence was found for positive associations with overall access to destinations/services, land use mix – destination diversity, parks/open space/recreational destinations, pedestrian-friendly features and easy access to building entrance (Table 3). Weaker evidence of positive associations was detected for street connectivity, access to food outlets, street lights and having benches/sitting facilities in the neighbourhood. Insufficient evidence of associations was found for the remaining 14 environmental attributes studied, while walkability and pollution were not investigated. The patterns of relationships were largely independent of the type of adjustment used in the meta-analyses (Additional file 5).

#### Moderators of associations

Age, sex, education [58] and chronic conditions [4] were investigated as moderators of within-neighbourhood walking only in single studies (Table 5). Land use mix – destination diversity and the presence of benches and sitting facilities were identified as significant correlates in older (75+ year olds) but not younger senior residents (65–74 year olds) [58]. Only one of 12 investigated moderating effects of sex was statistically significant and none were significant for education [58]. Having vs. not having a genitourinary or musculoskeletal disease was predictive of stronger associations with four out of 15 environmental attributes [4]. In contrast, not having vs. having visual impairment yielded stronger positive associations of neighbourhood aesthetics with weekly frequency and amounts of within-neighbourhood walking for transport.

### Neighbourhood environmental correlates of combined measures of walking and cycling for transport

The most frequently examined environmental features among the five articles reporting on combined measures of walking and cycling were parks/open spaces/recreational destinations, pedestrian-friendly features, greenery and aesthetically pleasing scenery, and traffic/pedestrian safety (Table 3). Evidence of positive associations was found for business/government/institutional/industrial destinations, walkability, shops/commercial destinations, food outlets and street lights. However, only the first of these characteristics was examined in more than one article. Insufficient evidence of associations was reported for 15 other neighbourhood attributes of which only two were investigated five or more times (greenery and aesthetically pleasing scenery and traffic/pedestrian safety). With the exception of traffic/pedestrian safety, the patterns of relationships were independent of the type of adjustment used in the meta-analyses (Additional file 5). All positive associations of traffic/pedestrian safety with walking and cycling were found when using objective measures of this environmental attribute [32], while perceived traffic/pedestrian safety yielded nil [59] or unexpected associations [47].

#### Moderators of associations

Only two articles studied moderators of associations of environmental attributes with combined measures of walking and cycling for transport. Both of these articles examined the moderating effect of area-level SES (Table 5) [34, 47]. Area-level household income did not moderate the associations with objectively-assessed walkability in the USA [34]. However, in a South African pilot sample, perceived pedestrian-friendly features were positively associated with this AT outcome only in high-SES areas [47]. Yet, the latter article did not conduct a formal analysis of moderators (Additional file 4).

### Neighbourhood environmental correlates of cycling for transport

Only two Belgian studies examined neighbourhood environmental correlates of cycling for transport in older adults [6, 25]. These studies examined a total of 12 environmental correlates (Table 3). A large population sample provided evidence for positive relations with access to public transport and shops/commercial services, and negative associations with urbanisation [25]. No differences in conclusions were observed when using different types of adjustment (Additional file 5).

#### Moderators of associations

A study examined area-level-household income as a moderator of the effects of walkability and found insufficient evidence of moderation (Table 5) [6]. Another large-scale Belgian study estimated the moderating effects of age, sex and urbanisation level on the associations of 12 perceived environmental attributes and cycling or not cycling for transport on a daily basis [25]. Complex age by sex, age by urbanisation and age by sex by urbanisation interaction effects were observed (detailed in Table 5).

### Neighbourhood environmental correlates of active travel (all outcomes combined)

The last set of columns in Table 3 provides a summary of the evidence of environmental correlates of all AT outcomes combined. Most of the evidence mirrored that of total walking for transport, which was the most frequently examined AT outcome. Differences were observed only with regards to four environmental characteristics. These were food outlets, business/government/institutional/industrial destinations, street lights and littering/vandalism/decay, whereby positive associations were found between the first three categories of environmental attributes with all AT outcomes but not with total walking for transport. The opposite was true for littering/vandalism/decay. Different types of adjustment for quality and sample size did not yield different conclusions (Additional file 5).

Different patterns of associations were found between objective and perceived measures of four environmental attributes and all AT outcomes (Table 4). Only perceived measures of residential density/urbanisation, land use mix – destination diversity and parks/open space/recreation destinations provided sufficient evidence of positive associations. The opposite held true for traffic/pedestrian safety.

## Discussion

In the last decade, the health, transportation and environmental sustainability sectors have become increasingly interested in finding ways to promote AT in older adults via environmental and policy interventions [10, 67–70]. This interest has been accompanied by an eight-fold increase in the number of articles on neighbourhood physical environmental correlates of older adults’ AT since the latest systematic review on this topic was published [19]. We have critically reviewed the empirical evidence and developed a novel meta-analytic procedure to statistically summarise and combine the evidence published in this millennium.

### Neighbourhood walkability and its components

We found very strong and consistent support for a positive association between objectively-assessed neighbourhood walkability and older adults’ total walking for transport. This relationship applied to older adults living in high- as well as low-income neighbourhoods [6, 34, 61]. Moderate-to-strong evidence of positive associations with total walking for transport also emerged for all individual components of walkability – namely, residential density, street connectivity and land use mix. As these three neighbourhood attributes have been also identified as independent contributors to walking for transport in younger adults (aged 18–65 years) from 14 cities across the globe [71], these findings speak in favour of the universal importance of walkability as a determinant of walking for transport across adulthood.

Among the various aspects of walkability, land use mix and access to destinations were more consistent correlates of older adults’ AT than residential density and street connectivity. This is understandable given that easy access to a variety of destinations is an essential component of AT, while residential density is usually a proxy for availability of destinations and services, and street connectivity is considered a facilitator rather than an essential factor for AT [72, 73]. Moreover, the effects of residential density may differ by AT outcome and geographical context. For example, a large population-based Belgian study reported a negative association between urbanisation level and older adults’ cycling for transport [25], which is in line with a multi-country study on adults aged 18–64 years [24]. Denser areas are typified by short distances to destinations and higher levels of traffic volume and hazards, possibly making cycling a less safe and less convenient mode of transport than walking.

We found no support for an association between residential density and within-neighbourhood walking. This may be attributable to all the evidence originating from an ultra-dense city (Hong Kong) [4, 58]. Increasing density in already highly dense areas may result in a decrease in walking for transport because distances to commercial, transportation and other services become very short and/or people may opt to ‘chain’ multiple trips to a single trip. In this regard, a multi-country study identified thresholds of 7,500-12,000 dwellings/km^2^ for objectively-assessed residential density after which the odds and frequency of adults’ walking for transport plateaued or decreased [71]. It is unknown whether these threshold values also apply to older adults. We need multi-country studies with comparable methodologies that would allow data pooling to be able to examine threshold effects and identity the optimal amount of density that supports walking and cycling for transport in older adults.

### Destinations that matter to older adults

While the availability of destinations is undoubtedly a crucial determinant of AT, it is plausible to assume that not all types of destinations are equally important to older adults [54, 57]. Among the nine categories of destinations examined in this review, public transport stops, shops/commercial destinations and parks/open spaces/recreational facilities were the most consistent correlates of AT, with most of the evidence coming from studies examining total walking for transport. Some, but weaker, evidence was also found for availability of food outlets and various business/institutional/industrial destinations with respect to all AT outcomes. In agreement with these findings, several travel diary studies revealed that shopping (including food purchases), errands, recreational activities and social activities were the most frequently reported purposes of all-mode and/or AT trips among older adults in Australia [74], Hong Kong [75] and the UK [53]. Also, shops, services, food outlets, transit stops and meeting points emerged as the most frequently visited destinations by Canadian [54] and Hong Kong older adults [75]. Good access to public transport is particularly important to older adults who do not live in destination-rich neighbourhoods [57] and have limited or no access to private travel options [22, 76].

### Pedestrian infrastructure and streetscape

We identified several important aspects of pedestrian infrastructure and streetscape that may promote AT in older adults. Pedestrian-friendly features, such as the presence of well-maintained footpaths and indoor places for walking, were found to be positively related to total as well as within-neighbourhood walking. The same held true for availability of benches/sitting facilities and having easy access to the residential building entrance, while the presence of street lights was associated with more AT in general. Overall, these findings mirror those of a review of qualitative investigations [77] and a recent experimental study [78] reporting that older adults considered the presence, quality and pedestrian-friendliness of footpaths as the most important set of micro-environmental features encouraging walking.

Older adults typically experience increasing levels of mobility limitations and fear of falling due to chronic health conditions, such as musculoskeletal diseases [79] and sensory impairments [80]. For this reason, they are more vulnerable to physically challenging environments than younger individuals. Being able to easily get out of one’s home into a safe, pedestrian-friendly environment that provides opportunities for resting (benches/sitting facilities) may be decisive factors for older adults’ engaging in AT [64]. While this review and meta-analysis support these contentions, it should be noted that, with the exception of pedestrian-friendly features, very few quantitative studies examined the effects of the above neighbourhood attributes. Also, findings on pedestrian-friendly features and total walking for transport were more heterogeneous than those related to walkability and its components. While availability of destinations is an essential determinant of AT, the importance of walking infrastructure for AT is likely to be dependent on the presence of relevant destinations and one’s functional mobility. The only study that examined the moderating effects of diagnosed chronic conditions affecting functional mobility on the associations of pedestrian infrastructure with walking for transport found stronger positive relationships in those with genitourinary and musculoskeletal diseases than in those without these diseases [4]. Moreover, the presence of benches/sitting facilities was related to within-neighbourhood walking for transport only in older Hong Kong elders (aged 75+ years) [58]. Finally, in an experimental study on environmental features supporting AT in Belgian older adults, Van Cauwenberg and colleagues observed that the availability of benches/sitting facilities was particularly important for those with functional mobility limitations and fear of falling [78].

### Other neighbourhood built environment characteristics

No evidence of associations with AT was found for most variables measuring neighbourhood aesthetics, cleanliness/order, traffic and crime-related safety. Weak evidence of a negative association was observed between the presence of litter, vandalism and decay and total walking for transport, suggesting that sometimes signs of neighbourhood disorder may discourage walking in older adults. Also, higher human and motorized traffic volumes tended to be positively correlated with walking for transport likely due to destination-rich, walkable areas also being heavily trafficked and visited by a large number of people. Some of these findings are in contrast to those reported in qualitative studies which emphasise the importance of safety [77]. The lack of consistent quantitative evidence of an effect of aesthetics and safety on AT in older adults mirrors the findings observed in younger adults [24, 81, 82]. There are several potential reasons responsible for these findings. It has been suggested that some individuals may engage in AT regardless of aesthetic or safety issues because they have no other option (e.g., they do not have access to public transport or own a car) [24, 76]. Measures of aesthetics and safety are typically generic and based on subjective evaluations that have different meaning and behavioural implications for different people [83, 84]. Neighbourhood aesthetics and safety may represent peripheral facilitators rather than essential determinants of AT and, for example, moderate the effects of access to destinations and services as observed in a study on Hong Kong older adults [57]. In this regard, Alfonzo hypothesised that the need for safety and aesthetics could influence walking only if the more important need of access to services is met [73]. Finally, most studies were conducted in high-income countries in which safety may not really be an issue, especially during the day when most of the AT is performed. In this respect, a multi-country study of adults including high- as well as mid-income, less safe countries (e.g., Mexico and Colombia) revealed that the positive association of safety from crime with PA was much stronger at the between-country than within-country level [85].

### Theoretical and methodological considerations

Research in the area of environmental correlates of AT has been inspired by Sallis et al.’s socio-ecological model of active living [23]. According to this model, physical environmental factors shape AT in a context-specific manner. Because walking and cycling for transport have different needs, they are likely influenced by different environmental features. Context specificity has also a geographical dimension according to which neighbourhood environmental attributes are hypothesised to impact on AT within and outside the neighbourhood in different ways [86, 87]. Yet, an analysis of the current evidence reveals that most studies focused on total walking for transport, irrespective of the location where it occurred. Also, more studies examined cycling in combination with walking than as a distinct AT outcome. Although from a public health perspective it is undoubtedly important to identify neighbourhood characteristics that are associated with higher overall levels of activity (e.g., total walking or total AT), from a behavioural perspective it is essential to understand how these characteristics impact on specific behaviours as this knowledge can more effectively guide environmental interventions.

Beside context specificity, another key tenet of the socio-ecological model of active living is the presence of interactions between factors shaping physical activity behaviour [23]. The effect of an environmental characteristic on AT is hypothesised to depend on (i.e., be moderated by) individual, social and other environmental factors. Human behaviour is a complex phenomenon that can seldom be accurately explained by simple additive models of exposures and individual characteristics. Yet, this review has revealed a dearth of findings on interactions. Eight of 11 studies that examined individual-level moderators of environment-AT associations found significant interaction effects of socio-demographic, health or psychosocial factors. Among the seven studies that investigated environmental moderators, three observed significant effects. Collectively, these findings suggest that different groups of older adults are likely to require different types of environmental interventions and the effectiveness of certain environmental modifications (e.g., increased access to public transport) may depend on other environmental conditions being met (e.g., high-quality pedestrian infrastructure). Solid knowledge of these moderating factors is necessary to effectively target the most vulnerable population subgroups of older adults and maximise the impact of environmental interventions. Whenever possible, this line of research needs to undertake theory-driven rather than purely exploratory analyses, given the large number of testable interactions effects arising from studies of AT adopting an ecological approach.

The presence of unexplored interaction effects may be one of the reasons for the heterogeneous findings observed in this review with respect to several categories of environmental factors. Another potential source of heterogeneity may be the different types of exposure measures. Self-report measures of land use mix and access to recreational destinations yielded stronger evidence of positive associations than did objective measures. The opposite was true for traffic/pedestrian safety with respect to AT, and for street connectivity with respect to walking for transport. Older adults who engage in AT are likely to be more aware of the availability of destinations within their neighbourhood than those who do not walk or cycle for transport. They are certainly more aware of the destinations that they visit (e.g., specific type of shop or food outlet). In contrast, objective measures of land use mix – destination diversity and destination availability may not be optimally operationalised and include a substantial number of places, services and/or land uses of little importance to older adults. In fact, not much is known about the optimal mix and number of destination types that might promote AT in this age group. Also, objective measures of destination availability are typically obtained for whole administrative areas [88–91] or home-centred buffers of various sizes [33, 92, 93], while self-report measures usually define a neighbourhood as an area within 10–20 min walk from a participant’s home [58, 94, 95]. Given that there is large inter-individual variability in functional mobility and walking speed among older adults [96], defining a neighbourhood using time as a parameter (as in self-report measures) may be more appropriate for this age group than defining it in terms of distance (as in objective measures of the environment). This could in part explain why this review found that objective distance-based measures of destination availability performed worse as correlates of AT than their self-report counterparts. It is interesting that, in contrast, objectively-assessed street connectivity and traffic/pedestrian safety were more consistently associated to AT outcomes than self-report measures of the same. It is possible that these neighbourhood attributes are more susceptible to individual response biases or more difficult to recall than destination availability due to them not being the main focus of attention during AT.

Apart from various methodological issues (e.g., confounding, insufficient statistical power, use of measures with varying metric characteristics), some of the heterogeneity in findings observed in this review may be also the result of limited within-study and large between-study variability in exposures. For example, as noted earlier, there is evidence that residential density may be curvilinearly related to walking for transport in adults [24, 71]. However, the predominately positive concave-downward relationship became apparent only after pooling data from different cities varying in residential density. This shape of relationship implies that data collected in low-to-mid density (e.g., <15,000 dwellings km^2^ [71]) areas may show a positive association between residential density and walking for transport, while studies conducted in high density areas (e.g., >15,000 [71] or >30,000 [58]) may yield nil or negative associations. In this review, we found this to be the case for studies based in the USA [22], Australia [97], eastern European countries [95], Belgium (low-to-medium density areas) [25], and Japan [35] and Hong Kong [58] (high density areas). To resolve discrepant findings potentially due to restricted variability in exposures and the presence of non-linear dose-repose relationships, pooled analyses of comparable data from geographical locations varying in exposure are necessary.

### Weaknesses of available evidence

One of the main weaknesses of the available evidence on physical environmental correlates of older adults’ AT is its cross-sectional nature. Cross-sectional studies cannot establish causality because they are affected by a range of threats to validity, residential self-selection being one of the most important even in good quality studies [98]. Only four of 42 reviewed articles attempted to address self-selection by including reasons for living in a specific neighbourhood or enjoyment of an active lifestyle as covariates in regression analyses [41, 54, 60, 61]. All four studies found evidence of positive associations between aspects of walkability and AT in older adults, indicating that the effects are unlikely to be entirely due to residential self-selection. Other reviews and studies that examined the potential effect of residential self-selection on PA [99] or related health outcomes [100] in other age groups reported similar patterns of findings and, in some instances, observed stronger associations in the expected direction after adjustment for self-selection [101]. Apart from more good-quality cross-sectional studies, this research field would benefit from stronger causal evidence based on well-conducted prospective and quasi-experimental studies, which may be challenging as the amount and rate of environmental changes is often insufficient during the life of a typical study to significantly impact on AT [99]. Prospective studies examining the effects of post-retirement relocation to less or more activity-friendly neighbourhoods on AT might address the likely lack of variability in environmental changes encountered in prospective studies of non-movers. However, studies of movers need to account for the fact that people who relocate to a new neighbourhood need a certain amount of time to become part of the community and familiarise with its physical environment. Also, prospective studies of movers raise selection bias concerns since movers may differ from non-movers in important ways that impact on their AT and adaptation to new environments.

Sampling bias was identified as a significant threat to validity in over 70% of the reviewed articles, with many studies reporting very low [22, 32, 34, 50, 53, 54, 63, 65, 66] or no information on response rates [47, 48, 50–52, 55, 64, 70, 89, 93, 95, 97]. Optimally, studies should not only report response rates but also provide an assessment of the representativeness of the sample and describe the implications of an identified or potential pattern of selection bias. It is particularly important to implement strategies aimed at maximising the response rate and participant retention. These have been extensively examined [102] and successfully employed in studies on neighbourhood correlates of AT in mid-aged adults [103]. Mailed surveys and face-to-face interviews appear to be associated with better response rates than web-based surveys and phone interviews in older adults [104].

Although most of the reviewed articles reported using validated measures of AT, they were all based on self-reports. AT is a form of incidental PA that may be more difficult to recall than leisure-time PA [86]. The use of Geographic Positioning System (GPS) devices with automated algorithms able to identify AT trips and modes (cycling versus walking) would help reduce measurement error and also clarify the extent to which neighbourhood environmental attributes impact on the geographical context of AT (e.g., walking within and outside the neighbourhood) [105, 106].

The analytical approaches adopted in the reviewed articles were often acceptable. Among the unacceptable practices were: (1) the failure to account for area-level clustering invalidating the standard errors of regression coefficients and, thus, conclusions [39]; (2) the failure to report exact p-values, standard errors or 95% confidence intervals of main effects; (3) the failure to probe significant interaction effects by estimating regression coefficients and their standard errors at meaningful values of the moderator; and (4) the tendency to transform continuous outcomes and exposures into categories. The latter issue has been shown not only to lead to loss of statistical power but also to increase residual confounding and the likelihood of spurious associations and interaction effects [40].

### Strengths and weaknesses of systematic review

There are several strengths to this systematic review and meta-analysis. We devised a conservative meta-analytic approach that allowed a quantitative synthesis of findings that did not rely on the here-inappropriate use of precise effect sizes. Both published peer-reviewed scientific and grey literatures were examined to address possible article selection biases. When possible, findings were presented by AT mode and environmental measure type (objective and self-reported). Quality assessment and sample size information was incorporated in the meta-analyses and sensitivity analyses were conducted to examine the effects of this information on the conclusions. Limitations include the inability to more accurately quantify associations due to large variability in exposures and outcomes; in some instances, not accounting for correlated findings extracted from the same study (e.g., multiple outcomes falling into the same AT category); the inability to examine the moderating effects of neighbourhood size on the strength of environment – AT associations due to studies not examining this issue (except for Etman et al.’s study, with no clear emerging patterns of associations [33]); and the exclusive focus on work published in English which possibly led to an over-representation of studies from developed countries.

### Unanswered questions and future research

**Fig. 2:**
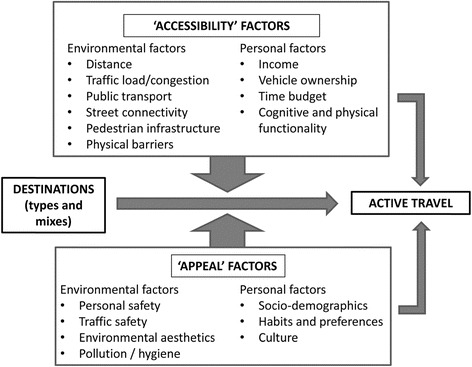
A proposed conceptual framework of AAA+ destinations: Available, Accessible, Appealing for Active travel in an Ageing population

Many substantive questions on the topic of this review remain unanswered. To guide future research, Fig. 2 depicts a proposed conceptual model that focuses on the most important neighbourhood feature for AT: destinations. Individuals are more likely to walk or cycle to/from places if they are within affordable distance from home. Yet, this review suggests that not all destinations are relevant to older adults. Shops, food outlets, commercial and government services, public transport stops and recreational facilities appear to be particularly important. However, little is known about the mix and density of types of shops, services and facilities that are necessary to optimally promote AT among older adults.

An optimal mix and density of destinations and services catering for older adults’ daily needs may be available in the community. However, whether older residents will wish to visit them depends on their appeal. Further, residents will walk or cycle to ‘appealing’ destinations and services only if they are sufficiently accessible by AT, and AT is a more appealing option than motorised transport. Figure 2 lists potential individual and environmental factors influencing access to destinations and the appeal of destinations and AT. These ‘accessibility’ and ‘appeal’ factors are hypothesised to moderate the associations of destination types and mixes with AT by determining one’s capacity to reach a destination by AT (‘accessibility’), one’s desire or need to visit a destination (destination ‘appeal’), and one’s preference to use AT (AT ‘appeal’). These factors are also postulated to independently influence AT, but only when destinations are available [73]. These propositions are in line with the moderating effects of sex [25, 35], age [25, 58, 63], health status [4, 34], pedestrian infrastructure and pedestrian/crime safety [57] found in a few studies included in this review, and are based on concepts from the socio-ecological models of active living [23], Webber et al.’s model of mobility in older adults [107], the transportation and planning literature [108] and time geography [108, 109]. Overall, the effects of potential moderators shown in Fig. 2 are largely unexplored and constitute an important component of the research agenda for future studies.

The mediating role of perceptions of the neighbourhood environment in explaining the effects of the objective environment on AT is another critical issue (not depicted in Fig. 2) that needs to be tackled in future studies. Only one of the 42 reviewed studies investigated this matter [51]. If, as suggested by this synthesis of evidence, perceptions of destinations are more powerful predictors of AT than objective measures, it is important to understand the extent to which and under what circumstances perceptions may be manipulated through environmental changes.

Another contested issue regards the definition of neighbourhood or size of the area that influences older adults’ AT behaviour. While self-report measures typically define a neighbourhood using a time metric (e.g., 15–20 min walk from home [110]), objective measures use distance (e.g., 400 m street-network buffer around the home). As the transportation literature suggests that most individuals are prepared to travel up to 20 min to discretionary destinations [69], it makes sense to define buffers around the home in terms of time. This is because the distance walked or cycled in a specific amount of time depends on one’s physical capacity and the environmental characteristics of the street network.

In general, the majority of studies on environmental correlates of older adults’ AT have been conducted in North America. There is a paucity of findings from other geographical regions. The same applies to cycling as a mode of transport, including the use of electric bicycles as a viable option for older adults with lower functional capacity [111]. Future studies need to address these knowledge gaps.

## Conclusions and implications for policy and practice

Although research on the neighbourhood physical environmental correlates of older adults’ AT has flourished in the last 5 years, more well-conducted studies across the globe are needed to establish the optimal profiles and thresholds of neighbourhood environmental attributes that support walking and cycling for transport in different groups of older adults. Acknowledging the limitations of the available evidence, this review suggests that in order to promote and support older adults’ AT, especially walking for transport, it is important that neighbourhoods be walkable. Specifically, older residents should be provided easy, within-walking-distance access to shops, public transport, recreational facilities and various commercial and institutional services through a network of well-maintained and safe footpaths with sufficient places to rest (i.e., benches). It is important that environmental enhancements be developed and implemented so that they are optimally matched to the context-specific needs (e.g., extant levels of density) and demographics (e.g., sex and age composition) of the community. Also, objective enhancements to the neighbourhood environment may need to be accompanied by efforts to raise awareness of these enhancements.

## Additional files

Additional file 1: PRISMA checklist. (DOC 62 kb)
Additional file 2: Table S1. Neighbourhood physical environment and active travel in older adults – study characteristics and findings. (DOCX 87 kb)
Additional file 3: Supplementary analytical example – computation of *p*-values for associations of food outlets with all active travel outcomes in older adults. (DOCX 26 kb)
Additional file 4: Table S2. Article quality assessment. (DOCX 28 kb)
Additional file 5: Table S3. Summary table of meta-analytic results of significance of associations of neighbourhood built environmental correlates of active travel outcomes in older adults by type of adjustment for article characteristics. (DOCX 35 kb)

## Abbreviations

AT: Active travel
CEPA: Council for the Environment and Physical Activity
GIS: Geographic Information Systems
GPS: Global Positioning System
NEWS: Neighborhood Environment Walkability Scale
PA: Physical activity
SD: Standard deviation
SES: Socio-economic status
UK: United Kingdom
USA: United States of America
