# Supplementary material for: The neighbourhood physical environment and active travel in older adults: a systematic review and meta-analysis
Source: Int J Behav Nutr Phys Act. 2017 Feb 6;14:15. doi: 10.1186/s12966-017-0471-5 (PMC5294838; doi:10.1186/s12966-017-0471-5)
Supplement: Additional file 2: Table S1. — Neighbourhood physical environment and active travel in older adults – study characteristics and findings. (DOCX 87 kb) [file 12966_2017_471_MOESM2_ESM.docx]

**Supplementary Table 1. Neighbourhood physical environment and active travel in older adults – study characteristics and findings**

| **Article number, [reference # in review] name & author**  **[Name of study, first author, publication year]** | **Participants**  **[Total sample size; urban, rural or mixed sample; response rate or proof of representativeness of sample; community dwellers or not; geographical location]** | **Study design**  **[Cross-sectional, longitudinal or experiment;**  **sampling method for clusters and individuals; stratification used by environment attributes; neighbourhood definition]** | **Covariates**  **[Covariates included in the analyses]** | **Outcome measures**  **[Active travel outcome measure; instrument; validity]** | **Environmental exposure variables**  **[Environmental variables, their type (objective vs perceived) and (in brackets) their classification into environmental categories (to assist compilation of summary table)]** | **Moderators**  **[Moderators examined and breakdown of sample size by qualitative moderator (e.g., sex; educational attainment)]** | **Analytical approach**  **[Analytical approach; adjustment for clustering; appropriateness (distributional assumptions; moderation analyses) and presentation]** | **Findings**  **Main effects or moderating effects (conclusion in red)** | **Comments**  **[Notes important for the assessment or interpretation of the study (if any)]** |
| --- | --- | --- | --- | --- | --- | --- | --- | --- | --- |
| **1A [93]**  Active Living Study  Boruff et al., 2012 | N = 325(urban)  Mean age: 77 years; 68% women  49% response rate (village)  46% response rate (person)  Retirement village dwellers  Perth, Australia | Cross-sectional  Cluster: purposive  Individuals: random and convenience  Stratification: walkability  Neighbourhood definition: variable (7 different types of buffers) | Age, sex, education, marital status, BMI, physical functioning | Transport walking (Yes/No) [CHAMPS; validated]  *Walk(YN)* | *Objective (each measure computed for 7 buffer types):*  % commercial land use (shops/commercial)  % institutional land use (business / government / institutional / industrial)  % recreational and park land use (parks / open space / recreation)  % industrial land use (business / government / institutional / industrial)  % residential land use (access/availability of services -> other)  % utilities / communications land use (access/availability of services -> other)  % other land use (access/availability of services -> other) | None | Generalized Estimating Equations with exchangeable correlation matrix | *Main effects with Walk(YN)*  % commercial land use:  All ORs p>.05 (shops/commercial **0**)  % institutional land use:  All ORs p>.05 (business / government / institutional / industrial **0**)  % recreational and park land use:  All ORs p>.05 (parks / open space / recreation **0**)  % industrial land use:  All ORs p>.05 (business / government / institutional / industrial **0**)  % residential land use:  All ORs p>.05 (access/availability of services -> other **0**)  % utilities / communications land use:  6 of 7 ORs p<.05 (access/availability of services -> other **- * 0.86**; **0 * 0.14**)  % other land use:  All ORs p>.05 (access/availability of services -> other **0**) | Use proportional weights as each measure is calculated using 7 different buffers. Note that there are multiple measures per environmental construct that need to be summed. |
| **2A [88]**  Active Living Study  Nathan et al., 2014 (Australasian Journal on Ageing) | N = 323 (urban)  Mean age: 77 years; 68% women  49% response rate (village)  46% response rate (person)  Retirement village dwellers  Perth, Australia | Cross-sectional  Cluster: purposive  Individuals: random and convenience  Stratification: walkability  Neighbourhood definition: retirement village;  400m street network buffer | Age, sex, physical functionality, sampling method, access to car, education | Transport walking (Yes/No) [CHAMPS; validated]  *Walk(YN)* | *Objective:*  Age care facility (health and aged care)  Clubhouse (entertainment)  Amenities (land use mix – destination diversity)  Recreational facilities (parks / open space / recreation)  Neighbourhood walkability (walkability) | None | Generalized Estimating Equations with exchangeable correlation matrix | *Main effects with Walk(YN)*  Age care facility:  OR p<.05 in single attribute model (health and aged care **-**)  Clubhouse:  OR p>.05 (entertainment **0**)  Amenities:  OR p>.05 (land use mix – destination diversity **0**)  Recreational facilities:  OR p>.05 (parks / open space / recreation **0**)  Neighbourhood walkability:  OR = 1.19 p<.05 (walkability **+**) | Report single attribute rather than fully-adjusted models because results not adjusted for self-selection. |
| **3A [60]**  Active Living Study  Nathan et al., 2014 (Environment & Behavior) | N = 323 (urban)  Mean age: 77 years; 68% women  49% response rate (village)  46% response rate (person)  Retirement village dwellers  Perth, Australia | Cross-sectional  Cluster: purposive  Individuals: random and convenience  Stratification: walkability  Neighbourhood definition: Retirement village; 10-15 min walk from village | Age, sex, education, physical functioning, neighbourhood walkability, sampling method | Transport walking (60+min/week Yes/No) [CHAMPS; validated]  *Walk(60+YN)* | *Perceived:*  Access to activity centre – village (entertainment)  Access to services – neighbourhood (access to destinations/services)  Proximate destinations – village + neighbourhood (access to destinations/services)  Infrastructure for walking – village + neighbourhood (pedestrian friendly features)  Aesthetics – village + neighbourhood (greenery and aesthetically pleasing scenery)  Safety from crime – village + neighbourhood (crime/personal safety)  Safety from traffic – village + neighbourhood (traffic/pedestrian safety)  Even gradient – village (barriers to walking/cycling)  Street connectivity – village (street connectivity)  Fewer physical barriers – neighbourhood (barriers to walking/cycling)  Orderliness – neighbourhood (crime/personal safety)  Age-appropriate infrastructure for walking – neighbourhood (pedestrian friendly features)  Traffic signal transition – neighbourhood (traffic/pedestrian safety) | None | Generalized Estimating Equations with exchangeable correlation matrix | *Main effects with Walk(60+YN)*  Access to activity centre – village:  OR p>.05 (entertainment **0**)  Access to services – neighbourhood:  OR p>.05 (access to destinations/services **0**)  Proximate destinations – village + neighbourhood:  OR p>.05 village; OR=1.93 p<.001 neighbourhood (access to destinations/services **0*0.5** village; **+*0.5** neighbourhood)  Infrastructure for walking – village + neighbourhood:  ORs p>.05 (pedestrian friendly features **0**)  Aesthetics – village + neighbourhood:  ORs p>.05 (greenery and aesthetically pleasing scenery **0**)  Safety from crime – village + neighbourhood:  ORs p>.05 (crime/personal safety **0**)  Safety from traffic – village + neighbourhood:  ORs p>.05 (traffic/pedestrian safety **0**)  Even gradient – village:  OR = 0.60 p<.05 (barriers to walking/cycling **+**)  Street connectivity – village:  OR p>.05 (street connectivity **0**)  Fewer physical barriers – neighbourhood:  OR p>.05 (barriers to walking/cycling **0**)  Orderliness – neighbourhood:  OR p>.05 (crime/personal safety **0**)  Age-appropriate infrastructure for walking – neighbourhood:  OR p>.05 (pedestrian friendly features **0**)  Traffic signal transition – neighbourhood:  OR p>.05 (traffic/pedestrian safety **0**) | Treat similar measures on village and neighbourhood environment as two buffers (assign fractional weights). Note that there are multiple measures per environmental construct that need to be summed.  Reporting fully adjusted models (with multiple environmental predictors) as these were adjusted for self-selection. |
| **4A [41]**  Active Living Study  Nathan et al., 2014 (Journal of Aging and Physical Activity) | N = 323 (urban)  Mean age: 77 years; 68% women  49% response rate (village)  46% response rate (person)  Retirement village dwellers  Perth, Australia | Cross-sectional  Cluster: purposive  Individuals: random and convenience  Stratification: walkability  Neighbourhood definition: 10-15 min walk from village;  400m street network buffer | Age, sex, physical functioning, education, sampling method | Transport walking (60+min/week Yes/No) [CHAMPS; validated]  *Walk(60+YN)* | Perceived attributes were included but are not reported here because they were reported in Nathan et al., 2014 (E&B) without adjustment for objective environment  Objective village-level attributes + walkability were included but are not reported here because they were reported in Nathan et al., 2014 (AJAG)  *Objective:*  Distance to local shop (shops/commercial)  Distance to supermarket (shops/commercial)  Distance to health service (health and aged-care)  Distance to entertainment facility (entertainment)  Distance to public transport (public transport)  Distance to public recreation area (parks / open space / recreation)  Traffic-volume exposure (human or motorised traffic volume)  Slope (barriers to walking/cycling) | None | Generalized Estimating Equations with exchangeable correlation matrix | *Main effects with Walk(60+YN)*  Distance to local shop:  OR negative p<.05 in single-attributes models (shops/commercial **+**)  Distance to supermarket:  OR negative p<.05 in single-attributes models (shops/commercial **+**)  Distance to health service:  OR negative p<.05 in single-attributes models (health and aged-care **+**)  Distance to entertainment facility:  OR p>.05 (entertainment **0**)  Distance to public transport:  OR negative p<.05 in single-attributes models (public transport **+**)  Distance to public recreation area:  OR p>.05 (parks / open space / recreation **0**)  Traffic-volume exposure:  OR = 1.46, p=.004 (human or motorised traffic volume +)  Slope:  OR p>.05 (barriers to walking/cycling **0**) | There are multiple measures per environmental construct that need to be summed. Do not report perceived measures as they were reported in Nathan et al., 2014 (Environment & Behavior) unadjusted for objective measures. Do not report objective village-level measures + walkability because reported in Nathan et al.2014 (Australasian Journal Ageing) unadjusted for neighbourhood-level measures. Reporting single-attribute models as self-selection shown not to be related to walking. |
| **5A [4]**  ALECS  Barnett et al., 2016 | N = 909 (urban)  65+ years; 66% women  71% response rate  Community dwellers  Hong Kong, China | Cross-sectional  Cluster: purposive  Individuals: convenience  Stratification: walkability; area-level SES  Neighbourhood definition: 10-20 min walk from home | Sex, age, education, car in household, living arrangements, marital status, type of housing, chronic conditions, type of recruitment centre | Frequency and amount of within-neighbourhood walking for transport [NWQ-CS; validated]  *FrNWalk*  *AmNWalk* | *Perceived:*  Land use mix – diversity (land use mix – destination diversity)  Land use mix access to services (access to destinations/services)  Proximity to public transport (public transport)  Proximity of recreational facilities (parks / open space / recreation)  Street connectivity (street connectivity)  Pedestrian infrastructure (pedestrian-friendly features)  Indoor places for walking (pedestrian-friendly features)  Physical barriers to walking (barriers to walking/cycling)  Easy access of residential entrance (easy access to building entrance)  Bridge/overpass connecting services (pedestrian-friendly features)  Dwelling density (residential density / urbanisation)  Crowdedness (human or motorised traffic volume)  Presence of people (crime/personal safety)  Social disorder/littering (littering/vandalism/ decay/ vacant buildings)  Crime (crime/personal safety)  Traffic and road hazards (traffic / pedestrian safety)  Traffic speed (traffic / pedestrian safety)  Fence separating sidewalk & traffic (traffic / pedestrian safety)  Aesthetics (greenery & aesthetically pleasing scenery)  Sitting facilities (benches / sitting facilities) | Diagnosed chronic condition  n (vision impair) = 527  n (musculoskeletal) = 445  n (genitourinary) = 200  n (hearing impair) = 164 | Generalized linear models with robust standard errors accounting for clustering (negative binomial variance and logarithmic link functions) | *Main effects*:  Land use mix – diversity:  FrNWalk: exp(b) = 1.22; p<.001 (land use mix – destination diversity **+**)  AmNWalk: exp(b) = 1.122; p=.022 (land use mix – destination diversity **+**)  Land use mix access to services:  FrNWalk: exp(b) = 1.37; p<.001 (access to destinations/services **+**)  AmNWalk: exp(b) = 1.38; p<.001 (access to destinations/services **+**)  Proximity to public transport:  FrNWalk: exp(b) = 0.96; p>.05 (public transport **0**)  AmNWalk: exp(b) = 1.01; p>.05 (public transport **0**)  Proximity of recreational facilities:  FrNWalk: exp(b) = 1.10; p=.006 (parks / open space / recreation **+**)  AmNWalk: exp(b) = 1.09; p=.042 (parks / open space / recreation **+**)  Street connectivity:  FrNWalk: exp(b) = 1.25; p<.001 (street connectivity **+**)  AmNWalk: exp(b) = 1.17; p=.015 (street connectivity **+**)  Pedestrian infrastructure:  FrNWalk: exp(b) = 1.31; p<.001 (pedestrian-friendly features **+**)  Indoor places for walking:  FrNWalk: exp(b) = 1.08; p=.017 (pedestrian-friendly feature **+**)  AmNWalk: exp(b) = 1.11; p=.014 (pedestrian-friendly feature **+**)  Physical barriers to walking:  FrNWalk: exp(b) = 0.81; p<.001 (barriers to walking/cycling **-**)  AmNWalk: exp(b) = 0.83; p=.012 (barriers to walking/cycling **-**)  Easy access of residential entrance:  FrNWalk: exp(b) = 1.05; p>.05 (easy access to building entrance **0**)  Bridge/overpass connecting services:  FrNWalk: exp(b) = 1.03; p>.05 (pedestrian-friendly feature **0**)  AmNWalk: exp(b) = 1.04; p>.05 (pedestrian-friendly feature **0**)  Dwelling density:  FrNWalk: exp(b) = 1.003; p=.004 (residential density / urbanisation **+**)  AmNWalk: exp(b) = 1.000; p>.05 (residential density / urbanisation **0**)  Crowdedness:  FrNWalk: exp(b) = 1.00; p>.05 (human or motorised traffic volume **0**)  AmNWalk: exp(b) = 0.97; p>.05 (human or motorised traffic volume **0**)  Presence of people:  FrNWalk: exp(b) = 1.26; p<.001 (crime/personal safety **+**)  AmNWalk: exp(b) = 1.22; p=.002 (crime/personal safety **+**)  Social disorder/littering:  FrNWalk: exp(b) = 0.97; p>.05 (littering/vandalism/ decay/ vacant buildings **0**)  AmNWalk: exp(b) = 0.96; p>.05 (littering/vandalism/ decay/ vacant buildings **0**)  Crime:  FrNWalk: exp(b) = 0.88; p=.004 (crime/personal safety **-**)  AmNWalk: exp(b) = 0.87; p=.017 (crime/personal safety **-**)  Traffic and road hazards:  FrNWalk: exp(b) = 0.91; p>.05 (traffic / pedestrian safety **0**)  Traffic speed:  FrNWalk: exp(b) = 0.91; p>.05 (traffic / pedestrian safety **0**)  AmNWalk: exp(b) = 0.97; p>.05 (traffic / pedestrian safety **0**)  Fence separating sidewalk & traffic:  FrNWalk: exp(b) = 1.02; p>.05 (traffic / pedestrian safety **0**)  AmNWalk: exp(b) = 1.05; p>.05 (traffic / pedestrian safety **0**)  Sitting facilities:  FrNWalk: exp(b) = 1.04; p>.05 (benches / sitting facilities **0**)  AmNWalk: exp(b) = 1.08; p=.033 (benches / sitting facilities **+**)  *Moderating effects:*  Land use mix access to services (do not count as same direction of effects):  Non-genitourinary - AmNWalk: exp(b) = 1.23; p<.001 (access to destinations/services +)  Genitourinary - AmNWalk: exp(b) = 2.23; p<.001 (access to destinations/services +)  Pedestrian infrastructure (do not count as same direction of effects):  Non-genitourinary - FrNWalk: exp(b) = 1.25; p=.004 (pedestrian-friendly features +)  Genitourinary - FrNWalk: exp(b) = 1.66; p<.001 (pedestrian-friendly features +)  Pedestrian infrastructure:  Non-genitourinary - AmNWalk: exp(b) = 1.17; p>.05 (pedestrian-friendly features **0*0.78**)  Genitourinary - AmNWalk: exp(b) = 1.81; p<.001 (pedestrian-friendly features **+* 0.22**)  Easy access of residential entrance:  Non-musculoskeletal - AmNWalk: exp(b) = 0.91; p>.05 (easy access to building entrance **0*0.51**)  Musculoskeletal - AmNWalk: exp(b) = 1.39; p=.018 (easy access to building entrance **+*0.49**)  Traffic and road hazards:  Non-genitourinary - AmNWalk: exp(b) = 0.95; p>.05 (traffic / pedestrian safety **0*0.78**)  Genitourinary - AmNWalk: exp(b) = 0.50; p<.001 (traffic / pedestrian safety **+*0.22**)  Aesthetics:  Non-vision impair – FrNWalk: exp(b) = 1.25; p=.004 (greenery & aesthetically pleasing scenery **+*0.42**)  Vision impair – FrNWalk: exp(b) = 0.95; p>.05 (greenery & aesthetically pleasing scenery **0*0.58**)  Non-vision impair – AmNWalk: exp(b) = 1.25; p=.004 (greenery & aesthetically pleasing scenery **+* 0.42**)  Vision impair – AmNWalk: exp(b) = 1.00; p>.05 (greenery & aesthetically pleasing scenery **0*0.58**) | Multiple measures of the same environmental attribute category yielding multiple hits. Fractional weights needed to account for moderating effects (subgroup of sample). Noted that moderating effects were adjusted for all significant environmental attributes. Do not report some moderating effects because those with and without a chronic condition showed similar associations. |
| **6A [53]**  BCC initiative – Rode Island Trial  King et al. 2006 | N = 109 (not reported)  Mean age = 75; 65% women  Response rate not reported  Community dwellers  Rhode Island, USA | Cross-sectional  Individual: convenience  Stratification: none  Neighbourhood definition: participant delimitation | Age, education | Transport walking (hr/wk) [CHAMPS; validated]  *AmWalk* | *Perceived:*  Residential density (residential density / urbanisation)  Land use mix – access (access to services/destinations)  Street connectivity (street connectivity)  Seeing and speaking with others when walking in the neighbourhood (crime/personal safety)  Loose or unattended dogs (crime/personal safety) | None | Linear regression | *Main effects with AmWalk:*  Residential density:  b p>05 (residential density / urbanisation **0**)  Land use mix – access:  b p>05 (access to services/destinations **0**)  Street connectivity:  b p>05 (street connectivity **0**)  Seeing and speaking with others when walking in the neighbourhood:  b p>05 (crime/personal safety **0**)  Loose or unattended dogs:  b p>05 (crime/personal safety **0**) | Note that there are multiple measures per environmental construct that need to be summed. |
| **7A [22]**  Belgian Aging Studies  Van Cauwenberg et al., 2012 | N = 48,879 (urban, semi-urban, rural)  65+ years; 56% women  65%-85% response rate  Community dwellers  Belgium (135 municipalities) | Cross-sectional  Cluster: municipality – 135 selected  Individual: random  Stratification: urbanisation (post-recruitment), age, sex  Neighbourhood definition: participant delimitation | Age, sex, urbanisation, functional limitations | Transport walking – frequency (daily YN)  Transport cycling – frequency (daily YN)  [not validated]  *Walk(daily YN)*  *Cycle(daily YN)* | *Objective:*  Urbanisation level (residential density / urbanisation)  *Perceived:*  Short distance to services (access to destinations/services)  Number of shops (shops / commercial)  Public transport (public transport)  Presence of public toilets (public toilets)  Presence of benches (benches / sitting facilities)  Presence of crossings (traffic/pedestrian safety)  Condition of sidewalks (pedestrian-friendly features)  Absence of high ramps (pedestrian-friendly features)  Traffic safety (traffic/pedestrian safety)  Feeling of unsafety (crime/personal safety)  Street lighting (street lights)  Absence of decay (littering / vandalism / decay)  Absence of noise (pollution)  Greenery (greenery and aesthetically pleasing scenery) | Sex  % (women) ~ 56%  % (men) ~44%  Age  % (<75 years) ~ 50%  % (≥75 years) ~ 50%  Urbanisation  % (urban) ~ 31.5%  % (semi-urban) ~ 37%  % (rural) ~ 31.5% | Multilevel logistic regression accounting for clustering | *Main effects with Walk(daily YN):*  *Perceived:*  Short distance to services:  OR = 1.19 p<.05 (access to destinations/services **+**)  Public transport:  OR = 1.13 p<.05 (public transport **+**)  Presence of public toilets:  ORs p>.05 (public toilets **0)**  Condition of sidewalks:  OR = 0.94 p<.05 (pedestrian-friendly features **-**)  Absence of high ramps  OR = 1.01 p>.05 (pedestrian-friendly features **0**)  Presence of crossings:  ORs p>.05 (traffic/pedestrian safety **0**)  Street lighting:  ORs p>.05 (street lights **0**)  Presence of benches:  ORs p>.05 (benches / sitting facilities **0**)  Feeling of unsafety:  OR = 0.93 p<.05 (crime/personal safety **+**)  Traffic safety:  ORs p>.05 (traffic/pedestrian safety **0**)  Absence of noise:  ORs = 0.73 – 0.87 p<.05 (pollution **+**)  Greenery:  OR = 1.01 p>.05 (greenery and aesthetically pleasing scenery **0**)  *Objective:*  Urbanisation level:  OR = 1.32 p<.05 (semi-urban vs urban); OR = 1.43 p<.05 (rural vs. urban) (residential density / urbanisation **+**)  *Main effects with Cycle(daily YN):*  *Perceived:*  Short distance to services:  OR p>.05 (access to destinations/services **0**)  Number of shops:  OR = 1.03 p<.05 (shops / commercial **+**)  Public transport:  OR = 1.08 p<.05 (public transport **+**)  Presence of public toilets:  ORs p>.05 (public toilets **0)**  Presence of crossings:  ORs = 0.96 to 1.08 p>.05 (traffic/pedestrian safety **0**)  Traffic safety:  ORs 0.74 to 0.84 p<.05 (traffic/pedestrian safety **-**)  Greenery:  OR = 1.01 p>.05 (greenery and aesthetically pleasing scenery **0**)  *Objective:*  Urbanisation level:  OR = 0.72 p<.05 (semi-urban vs urban); OR = 0.81 p>.05 (rural vs. urban) (residential density / urbanisation **-**)  *Moderating effects with Walk(daily YN):*  Number of shops:  Urban and semi-urban; rural ≥75 years: ORs ~ 1.02-1.05 p<.05 (shops / commercial **+*0.8425**)  Rural <75 years: OR=1.01 p>.05 (shops / commercial **0*0.1575**)  Absence of decay:  <75 years and men ≥75 years: ORs = 0.70 to 0.74 p<.05 (littering / vandalism / decay **+*0.72**)  women ≥75 years: OR= 0.82 p>.05 (littering / vandalism / decay **0*0.28**)  Absence of noise: (do not count as same direction of effects)  Age*Sex moderation: ORs = 0.73 – 0.87 p<.05 (pollution **+**)  *Moderating effects with Cycle(daily YN):*  Presence of public toilets: (do not count as same direction of effects)  Urbanisation*Sex moderation: ORs 0.86 to 1.09 p>.05 (public toilets **0**)  Age*Sex moderation: ORs = 0.93 to 1.06 p>.05 (public toilets **0)**  Presence of benches:  Urban, semi-urban, rural men: ORs = 0.93 to 1.09 p>.05 (benches / sitting facilities **0*0.8246**)  Rural women: ORs 1.21 p<.05 (benches / sitting facilities **+*0.1764**)  Age*Sex moderation: ORs = 0.96 to 1.10 p>.05 (do not count as same direction of effects)  Presence of crossings: (do not count as same direction of effects)  Age*Sex moderation: ORs = 0.96 to 1.08 p>.05 (traffic/pedestrian safety **0**)  Traffic safety: (do not count as same direction of effects)  Age*Sex moderation: ORs 0.74 to 0.84 p<.05 (traffic/pedestrian safety **-**)  Street lighting:  ≥75 years and men <75 years: ORs = 0.87 to 1.00 p>.05 (street lights **0*78**)  women <75 years: OR = 1.16 p<.05 (street lights **+*22**)  Absence of decay:  Rural, semi-urban, urban ≥75 years and urban women <75 years: ORs p>.05 (littering / vandalism / decay **0*0.9307**)  Urban men <75 years: OR = 0.73 p<.05 (littering / vandalism / decay **+*0.0693**)  Absence of noise:  Semi-urban, rural and urban ≥75 years, rural men <75 years, urban women <75 years: ORs p>.05 (pollution **0*0.8425**)  Urban men <75 years: OR = 0.78 p<.05 (pollution **+*0.0693**)  Rural women <75 years: OR = 1.27 p<.05 (pollution **-*0.0882**) | Note that there are multiple measures per environmental construct that need to be summed. Fractional weights needed as there are significant moderating effects. |
| **8A [61]**  BEPAS Seniors  Van Cauwenberg et al., 2016 | N = 391 (urban)  65+ years; 54% women  45% response rate  Community dwellers  Ghent, Belgium | Cross-sectional  Cluster: purposive  Individual: random  Stratification: walkability and median household income  Neighbourhood definition: statistical sectors | Age, sex, marital status, education, physical functioning, # motorised vehicles, residential self-selection | Transport walking (YN)  [IPAQ-L; validated]  *Walk(YN)* | *Objective:*  Walkability (walkability) | Area-level income  n (low) = 189  n (high) = 202 | Multilevel logistic regression accounting for clustering | *No moderating effects.*  *Main effects with Walk(YN):*  Walkability:  OR 3.6 p<.001 (walkability **+**) |  |
| **9A [6]**  BEPAS Seniors  Van Holle et al., 2014 | n = 438 (urban)  65+ years; 54% women  45% response rate  Community dwellers  Ghent, Belgium | Cross-sectional  Cluster: purposive  Individual: random  Stratification: walkability and median household income  Neighbourhood definition: statistical sectors | Age, sex, living situation, education, physical functioning | Transport walking (min/wk)  Transport cycling (min/wk)  [IPAQ-L; validated]  *AmWalk*  *AmCycle* | *Objective:*  Walkability (walkability) | Area-level income  n (low) ~ 219  n (high) ~ 219 | Multilevel linear regression accounting for clustering - squared root of outcome | *No moderating effects.*  *Main effects with AmWalk:*  Walkability:  b = 4.62 p<.001 (walkability **+**)  *Main effects with* *AmCycle*:  Walkability:  b = 0.18 p>.05 (walkability **0**) |  |
| **10A [111]**  BEPAS Seniors  Van Holle et al., 2016 | n = 438 (urban)  65+ years; 54% women  45% response rate  Community dwellers  Ghent, Belgium | Cross-sectional  Cluster: purposive  Individual: random  Stratification: walkability and median household income  Neighbourhood definition: statistical sectors | Age, sex, living situation, education, neighbourhood income | Transport walking (min/wk)  [IPAQ-L; validated]  *AmWalk* | *Perceived:*  Land use mix –diversity (land use mix – destination diversity)  Access to recreational facilities (park / open space / recreational)  Connectivity (street connectivity)  Physical barriers to walking (barriers to walking/cycling)  Infrastructure for walking (pedestrian-friendly features)  Aesthetics (greenery and aesthetically pleasing scenery)  Safety from crime (crime/personal safety)  Safety from motorized traffic speeding (traffic/pedestrian safety)  *Objective:*  Walkability (walkability) – not extracted as reported in Van Holle et al., 2010 | None | Multilevel linear regression accounting for clustering - squared root of outcome | *Main effects with AmWalk:*  Land use mix –diversity:  b = 1.29 p<.05 (land use mix – destination diversity **+**)  Access to recreational facilities:  b = 0.92 p<.001 (park / open space / recreational **+**)  Connectivity:  b = 1.76 p<.001 (street connectivity **+**)  Physical barriers to walking:  b = -0.37 p>.01 (barriers to walking/cycling **0**)  Infrastructure for walking:  b = 0.45 p>.05 (pedestrian-friendly features **0**)  Aesthetics:  b = 0.37 p>.05 (greenery and aesthetically pleasing scenery **0**)  Safety from crime:  b = -1.32 p<.001 (crime/personal safety **-**)  Safety from motorized traffic speeding:  b = 0.33 p>.05 (traffic/pedestrian safety **0**) | Objective walkability not extracted as reported in Van Holle et al., 2010 |
| **11A [91]**  CNDS  Mendes de Leon et al., 2009 | N = 4317 (urban)  65+ years; 61% women  79% response rate  Community dwellers  Chicago, USA | Cross-sectional  Cluster: convenience  Individual: all invited to participate  Stratification: none  Neighbourhood definition: census block group | Age, sex, education, income, marital status, years of residence in neighbourhood, medical conditions, season | Transport walking (min/2 weeks) [Health Interview Survey items; validation not reported]  *AmWalk* | *Perceived:*  Neighbourhood-level disorder (littering / vandalism / decay) | None | Multilevel regression models accounting for clustering – squared root transformation of outcome variable | *Main effects with AmWalk:*  Neighbourhood-level disorder:  b = -2.35 p = .01 (littering / vandalism / decay **-**) |  |
| **12A [33]**  ELANE  Etman et al., 2014 | N = 408 (urban)  65+ years; 53% women  44% response rate  Community dwellers  Spijkenisse, The Netherlands | Cross-sectional  Individual: random  Stratification: none  Neighbourhood definition: variable (4 buffer sizes: 400m, 800m, 1.2km, 1.6km) | Age, sex and frailty | Transportation walking (min/last 2 weeks) [LAPAQ; validated]  *AmWalk* | *Objective (audits; each measure computed for 4 buffer sizes):*  Aesthetics (aesthetics and cleanliness/order)  Functional features (pedestrian & cycling infrastructure; streetscape)  Safety (safety and traffic)  Destinations (land use mix – destination diversity) | Frailty  n (non-frail) = 307  n (frail) = 101 | Linear regression – log-transformed outcome variables and bootstrapped standard errors | *Main effects with FrActTrans:*  Aesthetics:  *b* positive and p<.05 for 800m and 1.2km only (aesthetics and cleanliness/order **0*0.5 +*0.5**)  Functional features:  *b* = 0.72 p=.01 for 400m only (pedestrian & cycling infrastructure; streetscape **0*0.75 +*0.25)**  Safety:  *b* p<.05 (safety and traffic **0*2**)  Destinations:  *b* positive and p<.01 for 400m and 800m only (land use mix – destination diversity **0*0.5 +*0.5**)  No significant moderating effects of frailty | Fractional weights needed as there are multiple measures (4 buffers) per environmental construct. |
| **13A [59]**  EpiFloripa Elderly  Corseuil et al., 2011 | N = 1652 (urban)  60+ years (M=70) ; 64% women  87% response rate  Community dwellers  Florianopolis, Brazil | Cross-sectional  Cluster: purposive  Individuals: random  Stratification: household income  Neighbourhood definition: 15 min walk from home | Age, sex, education, disability score, self-perceived health | Active transport (walking + cycling) – inactive, low-active, active [IPAQ-L; validated]  *ActTrans(3 cat)* | *Perceived:*  Sidewalk conditions (pedestrian-friendly features)  Green areas (greenery and aesthetically pleasing scenery)  Sidewalk steepness (barriers to walking/cycling)  Presence of hills (barriers to walking/cycling)  Presence of garbage (littering / vandalism / decay)  Open-air sewers (pollution)  Bikeways, trails (parks / open space / recreation)  Parks, athletic courts (parks / open space / recreation)  Traffic as a barrier for walking/cycling (traffic/pedestrian safety)  Drivers/ respect to pedestrians on crossings (traffic/pedestrian safety)  Smoke pollution (pollution)  Street lights at night (street lights)  Safe to walk during the day (crime/personal safety)  Safe to walk during the night (crime/personal safety) | None | Multinomial logistic regression accounting for clustering | *Main effects with ActTrans (3 cat)*:  Sidewalk conditions:  ORs p>.05 (pedestrian-friendly features **0**)  Green areas:  ORs p>.05 (greenery and aesthetically pleasing scenery **0**)  Sidewalk steepness:  ORs p>.05 (barriers to walking/cycling **0**)  Presence of hills:  ORs p>.05 (barriers to walking/cycling **0**)  Presence of garbage:  OR = 1.55 p<.05 for low-active (ref: active) (littering / vandalism / decay **-**)  Open-air sewers:  ORs p>.05 (pollution **0**)  Bikeways, trails:  ORs p>.05 (parks / open space / recreation **0**)  Parks, athletic courts:  OR = 1.75 for inactive (ref: active) if no parks p<.05 (parks / open space / recreation **+**)  Traffic as a barrier for walking/cycling:  ORs p>.05 (traffic/pedestrian safety **0**)  Drivers/ respect to pedestrians on crossings:  ORs p>.05 (traffic/pedestrian safety **0**)  Smoke pollution:  ORs p>.05 (pollution **0**)  Street lights:  ORs = 2.40+ p<.05 for low-active and inactive (ref: active) if none (street lights **+**)  Safe to walk during the day:  ORs p>.05 (crime/personal safety **0**)  Safe to walk during the night:  ORs p>.05 (crime/personal safety **0**) | Note that there are multiple measures per environmental construct that need to be summed. |
| **14A [95]**  EpiFloripa Elderly  Corseuil et al., 2016 (JAH) | N = 1637 (urban)  60+ years (M=70) ; 64% women  87% response rate  Community dwellers  Florianopolis, Brazil | Cross-sectional  Cluster: purposive  Individuals: random  Stratification: household income  Neighbourhood definition: 15 min walk from home | Age, sex, education, BMI, self-perceived health | Transport walking – inactive, low-active, active [IPAQ-L; validated]  *Walk(3 cat)* | *Perceived:*  Sidewalk conditions (pedestrian-friendly features)  Green areas (greenery and aesthetically pleasing scenery)  Sidewalk steepness (barriers to walking/cycling)  Presence of hills (barriers to walking/cycling)  Presence of garbage (littering / vandalism / decay)  Open-air sewers (pollution)  Bikeways, trails (parks / open space / recreation)  Parks, athletic courts (parks / open space / recreation)  Traffic as a barrier for walking/cycling (traffic/pedestrian safety)  Crosswalk (traffic/pedestrian safety)  Smoke pollution (pollution)  Street lights at night (street lights)  Safe to walk during the day (crime/personal safety)  Safe to walk during the night (crime/personal safety) | None | Multinomial logistic regression accounting for clustering | *Main effects with Walk(3 cat)*:  Sidewalk conditions:  ORs = 1.31+ p<.05 (pedestrian-friendly features **+**)  Green areas:  ORs p>.05 (greenery and aesthetically pleasing scenery **0**)  Sidewalk steepness:  ORs p>.05 (barriers to walking/cycling **0**)  Presence of hills:  ORs p>.05 (barriers to walking/cycling **0**)  Presence of garbage:  OR = 0.97- p<.05 (littering / vandalism / decay **-**)  Open-air sewers:  ORs p>.05 (pollution **0**)  Bikeways, trails:  ORs p>.05 (parks / open space / recreation **0**)  Parks, athletic courts:  OR = 1.60 for active (ref: inactive) p<.01 (parks / open space / recreation **+**)  Traffic as a barrier for walking/cycling:  ORs p>.05 (traffic/pedestrian safety **0**)  Crosswalk:  OR = 1.43 p<.05 (traffic/pedestrian safety **+**)  Smoke pollution:  ORs p>.05 (pollution **0**)  Street lights:  ORs = 2.30 p<.01 for active (ref: inactive) (street lights **+**)  Safe to walk during the day:  OR = 1.42 for low-active (ref: inactive) p<.05 (crime/personal safety **+**)  Safe to walk during the night:  ORs p>.05 (crime/personal safety **0**) | Note that there are multiple measures per environmental construct that need to be summed. |
| **15A [90]**  EpiFloripa Elderly  Corseuil et al., 2016 (JPAH) | N = 1667 (urban)  60+ years (M=70) ; 64% women  89% response rate  Community dwellers  Florianopolis, Brazil | Cross-sectional  Cluster: purposive  Individuals: random  Stratification: household income  Neighbourhood definition: census tract | Age, sex, education | Transport walking – Yes/NO [IPAQ-L; validated]  *Walk(YN)* | *Objective:*  Percentage streetlights (street lights)  Percentage paved streets (pedestrian-friendly features)  Percentage of sidewalks (pedestrian-friendly features)  Street density (street connectivity)  Street connectivity (street connectivity)  Land use mix (land use mix – destination diversity)  Public open spaces (YN) (parks / open space / recreation) | None | Multilevel logistic regression accounting for clustering | *Main effects with Walk(YN)*:  Percentage streetlights:  ORs p<.05 (street lights **0**)  Percentage paved streets:  ORs = 1.61+ p<.05 (pedestrian-friendly features **+**)  Percentage of sidewalks:  OR = 1.77 for high levels p<.05 (pedestrian-friendly features **+**)  Street density:  ORs p>.05 (street connectivity **0**)  Street connectivity:  OR = 1.85 for high levels p<.05 (street connectivity **+**)  Land use mix:  ORs p<.05 (land use mix – destination diversity **0**)  Public open spaces (YN):  ORs p<.05 (parks / open space / recreation **0**) | Note that there are multiple measures per environmental construct that need to be summed. |
| **16A [51]**  HK Elderly 1  Cerin et al., 2013 (HKMJ) | N = 484 (urban)  65+ years; 58% women  78% response rate  Community dwellers  Hong Kong, China | Cross-sectional  Cluster: purposive  Individuals: random  Stratification: walkability and SES  Neighbourhood definition: 10-15 min walk from home | Age, sex, education | Transport walking (min/week) [NWQ-CS; validated]  *AmNWalk* | *Perceived:*  Aesthetics (greenery and aesthetically pleasing scenery)  Social disorder/litter (littering / vandalism / decay)  Traffic speed (traffic/pedestrian safety)  Fence separating traffic from footpath (traffic/pedestrian safety)  Bridge/overpass connecting to services (pedestrian-friendly features)  Other NEWS attributes not extracted as reported in Cerin et al. 2014 (PHN) using a more appropriate analytical approach and for two outcome variables (frequency and weekly minutes of transport walking). This paper was a dissemination report for the funding agency. | None | Generalized linear mixed models accounting for clustering and positive skewness of transport walking | *Main effects with AmNWalk:*  Aesthetics:  r 0.01 p>.05 (greenery and aesthetically pleasing scenery **0**)  Social disorder/litter:  r 0.06 p>.05 (littering / vandalism / decay **0**)  Traffic speed:  r -0.05 p>.05 (traffic/pedestrian safety **0**)  Fence separating traffic from footpath:  r 0.01 p>.05 (traffic/pedestrian safety **0**)  Bridge/overpass connecting to services:  r -0.03 p>.05 (pedestrian-friendly features **0**) | Note that there are multiple measures per environmental construct that need to be summed. Other NEWS attributes not extracted as reported in Cerin et al. 2014 (PHN) using a more appropriate analytical approach and for two outcome variables (frequency and weekly minutes of transport walking). |
| **17A [57]**  HK elderly 1  Cerin et al. 2013 (IJBNPA) | N = 484 (urban)  65+ years; 58% women  78% response rate  Community dwellers  Hong Kong, China | Cross-sectional  Cluster: purposive  Individuals: random  Stratification: walkability and SES  Neighbourhood definition: 400m street-network buffer | Age, sex, education | Transport walking (min/week within-neighbourhood and overall) [NWQ-CS and IPAQ-LC; validated]  *AmNWalk*  *AmWalk* | *Objective (environmental audits):*  Health clinics/services – prevalence (health and aged-care)  Health clinics/services – diversity (health and aged-care)  Places of worship – prevalence (religious)  Places of worship – diversity (religious)  Public transit point – prevalence (public transport)  Recreational destinations – prevalence (parks / open space / recreation)  Recreational destinations – diversity (parks / open space / recreation)  Government/public facilities – prevalence (business / government / institutional / industrial)  Government/public facilities – diversity (business / government / institutional / industrial)  Entertainment destinations – prevalence (entertainment)  Entertainment destinations – diversity (entertainment)  Non-food retail and services – prevalence (shops / commercial)  Non-food retail and services – diversity (shops / commercial)  Food and grocery stores – prevalence (food outlets)  Food and grocery stores – diversity (food outlets)  Restaurants – prevalence (food outlets)  Restaurants – diversity (food outlets)  Sloping streets (barriers to walking/cycling)  Public facilities (public toilets) (benches sitting facilities) – [NB: *presented as one in article; but also conducted separate analyses*]  Good path conditions (pedestrian-friendly features)  Path obstructions (barriers to walking/cycling)  Stray animals (crime/personal safety)  Street lights (street lights)  Signs of crime/disorder (crime/personal safety)  Pedestrian safety (traffic/pedestrian safety) | Sloping street  Public facilities  Good path conditions  Path obstructions  Stray animals  Street lights  Signs of crime/disorder  Pedestrian safety  n average (mean) = 290  n high (+1SD) = 97  n low (-1SD) = 97 | Generalised linear mixed models accounting for clustering and positive skewness of transport walking outcomes | *Main effects*:  Health clinics/services – prevalence:  AmNWalk: exp(b)= 1.03 p>.05 (health and aged-care **0**)  AmWalk: exp(b) p>.05 (health and aged-care **0**)  Health clinics/services – diversity:  AmNWalk: exp(b)= 1.45 p=.048 (health and aged-care **+**)  AmWalk: exp(b) p>.05 (health and aged-care **0**)  Places of worship – prevalence:  AmNWalk: exp(b)=1.06 p>.05 (religious **0**)  AmWalk: exp(b) p>.05 (religious **0**)  Places of worship – diversity:  AmNWalk: exp(b)= 1.45 p=.031 (religious **+**)  AmWalk: exp(b) p>.05 (religious **0**)  Public transit point – prevalence:  AmNWalk: exp(b)=1.02 p>.05 (public transport **0**)  Recreational destinations – prevalence:  AmNWalk: exp(b) = 0.99 p>.05 (parks / open space / recreation **0**)  AmWalk: exp(b) p>.05 (parks / open space / recreation **0**)  Recreational destinations – diversity:  AmWalk: exp(b)= 1.13 p = .017 (parks / open space / recreation **+**)  Government/public facilities – prevalence:  AmNWalk: exp(b) = 1.00 p>.05 (business / government / institutional / industrial **0**)  AmWalk: exp(b) p>.05 (business / government / institutional / industrial **0**)  Government/public facilities – diversity:  AmNWalk: exp(b) = 0.92 p>.05 (business / government / institutional / industrial **0**)  AmWalk: exp(b) p>.05 (business / government / institutional / industrial **0**)  Entertainment destinations – prevalence:  AmNWalk: exp(b) = 1.00 p>.05 (entertainment **0**)  AmWalk: exp(b) p>.05 (entertainment **0**)  Entertainment destinations – diversity:  AmWalk: exp(b) p>.05 (entertainment **0**)  Non-food retail and services – prevalence:  AmWalk: exp(b) p>.05 (shops / commercial **0**)  Non-food retail and services – diversity:  AmNWalk: exp(b) = 1.08 p>.05 (shops / commercial **0**)  AmWalk: exp(b) p>.05 (shops / commercial **0**)  Food and grocery stores – prevalence:  AmWalk: exp(b) p>.05 (food outlets **0**)  Food and grocery stores – diversity:  AmNWalk: exp(b) = 1.10 p>.05 (food outlets **0**)  AmWalk: exp(b) p>.05 (food outlets **0**)  Restaurants – prevalence:  AmNWalk: exp(b) = 1.01 p<.001 (food outlets **+**)  AmWalk: exp(b) p>.05 (food outlets **0**)  Restaurants – diversity:  AmNWalk: exp(b) = 1.12 p>.05 (food outlets **0**)  AmWalk: exp(b) p>.05 (food outlets **0**)  Sloping streets:  AmNWalk: exp(b) p>.05 (barriers to walking/cycling **0**)  AmWalk: exp(b) p>.05 (barriers to walking/cycling **0**)  Public facilities:  AmNWalk: exp(b) p>.05 (public toilets **0**)  AmNWalk: exp(b) p>.05 (benches sitting facilities **0**)  AmWalk: exp(b) p>.05 (public toilets **0**)  AmWalk: exp(b) p>.05 (benches sitting facilities **0**)  Good path conditions:  AmNWalk: exp(b) p>.05 (pedestrian-friendly features **0**)  AmWalk: exp(b) p>.05 (pedestrian-friendly features **0**)  Path obstructions:  AmNWalk: exp(b) p>.05 (barriers to walking/cycling **0**)  AmWalk: exp(b) p>.05 (barriers to walking/cycling **0**)  Stray animals:  AmNWalk: exp(b) = 0.97 p=.05 (crime/personal safety **+**)  AmWalk: exp(b) p>.05 (crime/personal safety **0**)  Street lights:  AmNWalk: exp(b) = 1.02 p<.001 (street lights **+**)  AmWalk: exp(b) p>.05 (street lights **0**)  Signs of crime/disorder:  AmNWalk: exp(b) = 1.28 p=.015 (crime/personal safety **-**)  AmWalk: exp(b) = 1.22 p=.039 (crime/personal safety **-**)  Pedestrian safety:  AmNWalk: exp(b) p>.05 (traffic/pedestrian safety **0**)  AmWalk: exp(b) p>.05 (traffic/pedestrian safety **0**)  *Moderating effects:*  Public transit point – prevalence:  No stray animals – AmWalk: exp(b) = 1.03 p=.006 (public transport **+*0.20**)  High stray animals – AmWalk: exp(b) p>.05 (public transport **0*0.20**)  Average stray animals – AmWalk: exp(b) p>.05 (public transport **0*0.60**)  Recreational destinations – diversity:  No signs of crime/disorder - AmNWalk: exp(b)= 1.19 p = .010 (parks / open space / recreation **+*0.10**)  High signs of crime/disorder - AmNWalk: exp(b)= 0.84 p > .05 (parks / open space / recreation **0*0.10**)  Average signs of crime/disorder - AmNWalk: exp(b)= 1.16 p = .008 (parks / open space / recreation **+*0.30**)  No stray animals - AmNWalk: exp(b)= 1.23 p = .009 (parks / open space / recreation **+*0.10**)  High stray animals - AmNWalk: exp(b)= 0.71 p > .05 (parks / open space / recreation **0*0.10**)  Average stray animals - AmNWalk: exp(b)= 1.16 p = .008 (parks / open space / recreation **+*0.30**)  Entertainment destinations – diversity:  No signs of crime/disorder - AmNWalk: exp(b)= 1.35 p = .002 (entertainment **+*0.20**)  High signs of crime/disorder - AmNWalk: exp(b)= 0.77 p > .05 (entertainment **0*0.20**)  Average signs of crime/disorder - AmNWalk: exp(b)= 1.08 p > .05 (entertainment **+*0.60**)  Non-food retail and services – prevalence:  Low path obstructions - AmNWalk: exp(b)= 1.00 p > .05 (shops/commercial **0*0.10**)  High path obstructions - AmNWalk: exp(b)= 1.01 p = .008 (shops/commercial **+*0.10**)  Average path obstruction - AmNWalk: exp(b)= 1.01 p = .047 (shops/commercial **+*0.30**)  No sloping streets - AmNWalk: exp(b)= 1.02 p = .032 (shops/commercial **+*0.10**)  High sloping streets - AmNWalk: exp(b)= 1.00 p > .05 (shops/commercial **0*0.10**)  Average sloping streets - AmNWalk: exp(b)= 1.01 p = .047 (shops/commercial **+*0.30**)  Food and grocery stores – prevalence:  Low path obstructions - AmNWalk: exp(b)= 1.02 p < .001 (food outlets **+*0.10**)  High path obstructions - AmNWalk: exp(b)= 1.00 p > .05 (food outlets **0*0.10**)  Average path obstruction - AmNWalk: exp(b)= 1.02 p = .044 (food outlets **+*0.30**)  No sloping streets - AmNWalk: exp(b)= 1.02 p = .032 (food outlets **+*0.10**)  High sloping streets - AmNWalk: exp(b)= 1.01 p > .05 (food outlets **0*0.10**)  Average sloping streets - AmNWalk: exp(b)= 1.02 p = .044 (food outlets **+*0.30**) | Some moderating effects with fractional weights. Note that there are multiple measures per environmental construct that need to be summed. |
| **18A [58]**  HK Elderly 1  Cerin et al., 2014 (PHN) | N = 484 (urban)  65+ years; 58% women  78% response rate  Community dwellers  Hong Kong, China | Cross-sectional  Cluster: purposive  Individuals: random  Stratification: walkability and SES  Neighbourhood definition: 10-15 min walk from home | Age, sex, education | Transport walking (frequency/week and min/week within-neighbourhood and overall) [NWQ-CS and IPAQ-LC; validated]  *FrNWalk*  *AmNWalk*  *FrWalk*  *AmWalk* | *Perceived:*  Residential density (residential density / urbanisation)  Land use mix – diversity (land use mix – destination diversity)  Land use mix - access to shops (access to destinations/services)  Access to public transport (public transport)  Street connectivity (street connectivity)  Physical barriers to walking (barriers to walking/cycling)  Infrastructure for walking (pedestrian-friendly features)  Indoor places for walking (pedestrian-friendly features)  Presence of people (crime/personal safety)  Crowdedness (human or motorised traffic volume)  Traffic and road hazards (traffic / pedestrian safety)  Crime (crime/personal safety)  Easy access of residential entrance (easy access to building entrance)  Sitting facilities (benches / sitting facilities) | Age  n (65-74 years) = 324  n (75 + years) = 160  Sex  n (women) = 283  n (men) = 201  Education  Up to primary = 295  Secondary or above = 189 | Generalised additive mixed models accounting for clustering and positive skewness of transport walking outcomes | *Main effects*:  Residential density:  FrNWalk: exp(b) = 1.00; p>.05 (residential density / urbanisation **0**)  AmNWalk: exp(b) = 1.00; p>.05 (residential density / urbanisation **0**)  FrWalk: exp(b) = 1.00; p>.05 (residential density / urbanisation **0**)  AmWalk: exp(b) = 1.001; p<.05 (residential density / urbanisation **+**)  Land use mix – diversity:  FrWalk: exp(b) = 1.05; p<.001 (land use mix – destination diversity **+**)  AmWalk: exp(b) = 1.21; p<.01 (land use mix – destination diversity **+**)  Land use mix - access to shops:  FrNWalk: exp(b) = 1.24; p<.05 (access to destinations/services **+**)  AmNWalk: exp(b) = 1.25; p<.001 (access to destinations/services **+**)  FrWalk: exp(b) = 1.05; p<.01 (access to destinations/services **+**)  AmWalk: exp(b) = 1.05; p>.05 (access to destinations/services **0**)  Access to public transport:  FrNWalk: exp(b) = 1.21; p>.05 (public transport **0**)  AmNWalk: exp(b) = 0.99; p>.05 (public transport **0**)  FrWalk: exp(b) = 1.10; p<.01 (public transport **+**)  AmWalk: exp(b) = 1.25; p<.05 (public transport **+**)  Street connectivity:  FrNWalk: exp(b) = 1.04; p>.05 (street connectivity **0**)  AmNWalk: exp(b) = 0.92; p>.05 (street connectivity **0**)  FrWalk: exp(b) = 1.02; p>.05 (street connectivity **0**)  AmWalk: exp(b) = 0.98; p>.05 (street connectivity **0**)  Physical barriers to walking:  FrNWalk: exp(b) = 1.16; p<.05 (barriers to walking/cycling **+**)  AmNWalk: exp(b) = 1.16; p>.05 (barriers to walking/cycling **0**)  FrWalk: exp(b) = 1.00; p>.05 (barriers to walking/cycling **0**)  AmWalk: exp(b) = 0.93; p>.05 (barriers to walking/cycling **0**)  Infrastructure for walking:  FrNWalk: exp(b) = 1.38; p<.05 (pedestrian-friendly features **+**)  AmNWalk: exp(b) = 1.38; p<.01 (pedestrian-friendly features **+**)  FrWalk: exp(b) = 1.12; p<.01 (pedestrian-friendly features **+**)  AmWalk: exp(b) = 1.29; p>.05 (pedestrian-friendly features **0**)  Indoor places for walking:  FrNWalk: exp(b) = 1.00; p>.05 (pedestrian-friendly features **0**)  AmNWalk: exp(b) = 0.98; p>.05 (pedestrian-friendly features **0**)  FrWalk: exp(b) = 1.03; p>.05 (pedestrian-friendly features **0**)  AmWalk: exp(b) = 1.03; p>.05 (pedestrian-friendly features **0**)  Presence of people:  FrNWalk: exp(b) = 1.16; p>.05 (crime/personal safety **0**)  AmNWalk: exp(b) = 0.99; p>.05 (crime/personal safety **0**)  FrWalk: exp(b) = 1.03; p>.05 (crime/personal safety **0**)  AmWalk: exp(b) = 1.10; p>.05 (crime/personal safety **0**)  Crowdedness:  FrNWalk: exp(b) = 1.16; p<.05 (human or motorised traffic volume **+**)  AmNWalk: exp(b) = 1.23; p<.05 (human or motorised traffic volume **+**)  FrWalk: exp(b) = 1.00; p>.05 (human or motorised traffic volume **0**)  AmWalk: exp(b) = 1.11; p<.05 (human or motorised traffic volume **+**)  Traffic and road hazards:  FrNWalk: exp(b) = 1.22; p<.05 (traffic / pedestrian safety **-**)  AmNWalk: exp(b) = 1.26; p<.01 (traffic / pedestrian safety **-**)  FrWalk: exp(b) = 1.00; p>.05 (traffic / pedestrian safety **0**)  AmWalk: exp(b) = 1.09; p>.05 (traffic / pedestrian safety **0**)  Crime:  FrNWalk: exp(b) = 0.85; p>.05 (crime/personal safety **0**)  AmNWalk: exp(b) = 1.21; p>.05 (crime/personal safety **0**)  FrWalk: exp(b) = 0.97; p>.05 (crime/personal safety **0**)  Easy access of residential entrance:  FrNWalk: exp(b) = 1.43; p<.001 (easy access to building entrance **+**)  AmNWalk: exp(b) = 1.41; p<.01 (easy access to building entrance **+**)  FrWalk: exp(b) = 1.05; p<.001 (easy access to building entrance **+**)  AmWalk: exp(b) = 1.14; p<.001 (easy access to building entrance **+**)  Sitting facilities:  FrNWalk: exp(b) = 1.28; p<.001 (benches / sitting facilities **+**)  FrWalk: exp(b) = 1.03; p<.01 (benches / sitting facilities **+**)  AmWalk: exp(b) = 1.10; p<.001 (benches / sitting facilities **+**)  *Moderating effects:*  Land use mix - diversity:  65-74 years - FrNWalk: exp(b) p>.05 (land use mix – destination diversity **0*0.67**)  75+ years - FrNWalk: exp(b)=1.47 p<.001 (land use mix – destination diversity **+*0.33**)  65-74 years - AmNWalk: exp(b) p>.05 (land use mix – destination diversity **0*0.67**)  75+ years - AmNWalk: exp(b)=1.46 p<.01 (land use mix – destination diversity **+*0.33**)  Crime:  Men - AmWalk: exp(b) p>.05 (crime/personal safety **0*0.37**)  Women - AmWalk: exp(b) = 1.37; p<.001 (crime/personal safety **-*0.63**)  Sitting facilities:  65-74 years - AmNWalk: exp(b) p>.05 (benches / sitting facilities **0*0.67**)  75+ years - AmNWalk: exp(b)=1.51 p<.001 (benches / sitting facilities **+*0.33**) | Some moderating effects with fractional weights. Note that there are multiple measures per environmental construct that need to be summed. 4 transport walking outcomes. |
| **19A [92]**  MOBILIZE Boston Study  Procter-Gray et al., 2015 | N = 745 (urban)  Mean age ~ 78; 64% women  68% response rate  Community dwellers  Boston, USA | Cross-sectional  Cluster: purposively selected communities  Households: household randomly selected within study region  Individual: all eligible older adults within household  Stratification: none  Neighbourhood definition: community | Age, sex, ethnicity, education, health-related variables | Transportation walking (YN) [not validated]  *Walk(YN)* | *Objective:*  Distance to bus stop (public transport)  Distance to subway (public transport)  Distance to hospital (health and aged-care)  Distance to shopping center or mall (shops / commercial)  Distance to post office (business / government / institutional / industrial)  Distance to public park (>1acre) (park / open space / recreational)  Distance to grocery/convenience store (food outlets)  Distance to town hall (business / government / institutional / industrial)  Distance to public library (business / government / institutional / industrial) | None | Logistic regression  (neighbourhood-level clustering not accounted for) | *Main effects with Walk(YN):*  Distance to bus stop:  OR = 0.25 p<.01 (public transport **+**)  Distance to subway:  OR = 0.69 p<.01 (public transport **+**)  Distance to hospital:  OR = 0.69 p<.01 (health and aged-care **+**)  Distance to shopping center or mall:  OR = 0.85 p<.01 (shops / commercial **+**)  Distance to post office:  OR = 0.64 p<.01 (business / government / institutional / industrial **+**)  Distance to public park:  OR = 0.41 p<.01 (park / open space / recreational **+**)  Distance to grocery/convenience store:  OR = 0.26 p<.01 (food outlets **+**)  Distance to town hall:  OR = 0.85 p<.01 (business / government / institutional / industrial **+**)  Distance to public library:  OR = 0.65 p<.01 (business / government / institutional / industrial **+**) | Note that there are multiple measures per environmental construct that need to be summed |
| **20A [89]**  Montreal’s Household Travel Survey  Moniruzzaman et al., 2013 | N = unknown (31,631 trips; N likely > 15,000) (urban)  Mean age likely >65 years; % women not reported  Response rate not reported  Community dwellers  Montreal, Canada | Cross-sectional  Individual: random  Stratification: none  Neighbourhood definition: dissemination area (census) | Age, sex, driver’s licence, occupation, income, marital status | Walking trip in 1 weekday (vs. car trip) [diary from Household Travel Survey; validated]  *Walk(YN)* | *Objective:*  Intersection density (street connectivity)  Street density (street connectivity)  Land use mix (land use mix – destination diversity) | None | Multinomial logit regression (clustering at the census-area level not accounted for) | *Main effects with Walk(YN):*  Intersection density:  b = 0.00 p>.05 (street connectivity **0**)  Street density:  b = 0.02 p = .008 (street connectivity **+**)  Land use mix:  b = 0.18 p = .022 (land use mix – destination diversity +) | Note that there are multiple measures per environmental construct that need to be summed. |
| **21A [70]**  Montreal’s Household Travel Survey  Moniruzzaman et al., 2015 | N = unknown (31,631 trips; N likely > 15,000) (urban)  Mean age likely >65 years; % women not reported  Response rate not reported  Community dwellers  Montreal, Canada | Cross-sectional  Individual: random  Stratification: none  Neighbourhood definition: dissemination area (census) | Age, sex, driver’s licence, occupation, income, marital status, vehicle ownership | Walking trips (frequency/day in 3 categories) [diary from Household Travel Survey; validated]  *FrWalk(3 cat)* | *Objective:*  Population density (residential density / urbanisation)  Employment density (business / government / institutional / industrial)  Network density (street connectivity)  Land-use mix (land use mix – destination diversity)  Activity locations (land use mix – destination diversity)  Distance to nearest pharmacy (health and aged-care)  Distance to nearest health facility (health and aged-care)  Distance to nearest bank (business / government / institutional / industrial)  Distance to nearest grocery (food outlet)  Distance to nearest library (business / government / institutional / industrial) | None | Trivariate ordered probit model (clustering at the census-are level not accounted for) | *Main effects with FrWalk(3 cat):*  Population density:  b p>.05 (residential density / urbanisation **0**)  Employment density:  b = -0.35 for medium density b = -0.11 for high density (business / government / institutional / industrial **-**)  Network density:  b p>.05 (street connectivity **0**)  Land-use mix:  b p>.05 (land use mix – destination diversity **0**)  Activity locations:  b = 1.19 p<.001 (land use mix – destination diversity **+**)  Distance to nearest pharmacy:  b p>.05 (health and aged-care **0**)  Distance to nearest health facility:  b p>.05 (health and aged-care **0**)  Distance to nearest bank:  b = -0.16 p<.001 (business / government / institutional / industrial **+**)  Distance to nearest grocery:  b p>.05 (food outlet **0**)  Distance to nearest library:  b p>.05 (business / government / institutional / industrial **0**) | Note that there are multiple measures per environmental construct that need to be summed. |
| **22A [52]**  NASH  King, 2008 | N = 190 (urban)  65+ years; 57% women  Response rate not reported  Community dwellers  Denver, USA | Cross-sectional  Cluster: purposive  Individual: systematic  Stratification: crime, household income, walkability, demographics  Neighbourhood definition: statistical neighbourhood | Age, sex, income, chronic disease score | Transportation walking (times/wk) [CHAMPS; validated]  *FrWalk* | *Objective (audits):*  Route continuity (pedestrian-friendly features)  Sidewalk width (pedestrian-friendly features)  Sidewalk maintenance (pedestrian-friendly features)  Traffic buffer (traffic/pedestrian safety)  Curb cuts (traffic/pedestrian safety)  Pedestrian signals (traffic/pedestrian safety)  Crosswalks (traffic/pedestrian safety)  Litter (litter / vandalism / decay)  Graffiti (litter / vandalism / decay)  Yard maintenance (greenery and aesthetically-pleasing scenery)  % non-residential destinations (land use mix – destination diversity)  Retail density (shops / commercial)  Services density (access to destination/services)  Exercise opportunities (park / open space / recreational)  Tennis courts (park / open space / recreational) | None | Multilevel linear regression accounting for clustering | *Main effects with FrWalk:*  Route continuity:  b = 0.25 p>.05 (pedestrian-friendly features **0**)  Sidewalk width:  b = 0.45 p>.05 (pedestrian-friendly features **0**)  Sidewalk maintenance:  b = -0.25 p>.05 (pedestrian-friendly features **0**)  Traffic buffer:  b = 0.16 p>.05 (traffic/pedestrian safety **0**)  Curb cuts:  b = 1.01 p<.01 (traffic/pedestrian safety **+**)  Pedestrian signals:  b = -2.73 p<.001 (traffic/pedestrian safety **-**)  Crosswalks:  b = 2.86 p<.001 (traffic/pedestrian safety **+**)  Litter:  b = -0.32 p>.05 (litter / vandalism / decay **0**)  Graffiti:  b = -0.75 p<.05 (litter / vandalism / decay **-**)  Yard maintenance:  b = -0.04 p>.05 (greenery and aesthetically-pleasing scenery **0**)  % non-residential destinations:  b = 2.77 p>.05 (land use mix – destination diversity **0**)  Retail density:  b = 0.05 p<.05 (shops / commercial **+**)  Services density:  b = 0.06 p>.05 (access to destination/services **0**)  Exercise opportunities:  b = -0.05 p>.05 (park / open space / recreational **0**)  Tennis courts:  b = -5.05 p>.05 (park / open space / recreational **0**) | Note that there are multiple measures per environmental construct that need to be summed. |
| **23A [54]**  Project OPAL  Davis et al., 2011 | N = 214 (urban)  70+ years; 49% women  21% response rate (representative)  Community dwellers  Bristol, UK | Cross-sectional  General medical practices: purposive  Individual: random  Stratification: areas by proximity to shops and deprivation index  Neighbourhood definition: 5-min walk from home | None | Active transport (trips/week) [daily trip logs; validation not reported]  *FrActTrans* | *Perceived:*  Amenities within 5-min walk (land use mix – destination diversity) | None | ANOVA – log transformed outcome variable | *Main effects with FrActTrans:*  Amenities within 5-min walk:  F ratio p=.002 indicative of a positive association (land use mix – destination diversity **+**) |  |
| **24A [64]**  SCAMOB Project  Tsai et al., 2013 | n = 657 (urban)  75+ years; 75% women  Response not reported  Community dwellers  Jyväskylä, Finland | Cross-sectional  Individual: convenience / volunteers  Stratification: none  Neighbourhood definition: not reported | Age, sex, health variables | Transportation walking (amount: low, moderate high) [not validated]  *Walk(3 cat)* | *Perceived:*  Traffic (traffic/pedestrian safety)  Terrain (barriers to walking/cycling)  Distances (land use mix – destination diversity)  Entrance (easy access to building entrance) | Living arrangement  n (alone) = 381  n (with others) = 276 | Multinomial regression analyses | *Main effects with Walk(3 cat):*  Traffic:  ORs p>.05 (traffic/pedestrian safety **0**)  Terrain:  ORs p>.05 (barriers to walking/cycling **0**)  Distances:  ORs ~ 7.50 p<.001 for low vs high walking (land use mix – destination diversity **+**)  *Moderating effects:*  Entrance:  Living alone - OR = 8.76 p<.001 for low vs high; OR = 2.13 p<.05 for moderate vs high (easy access to building entrance **+*0.56**)  Living with others - ORs p>.05 (easy access to building entrance **0*0.44**) |  |
| **25A [94]**  Singapore Longitudinal Aging Study  Nyunt et al., 2015 | N = 402 (urban)  Mean age: 69 years; 61% women  Response rate not reported  Community dwellers  Singapore | Cross-sectional  Cluster: convenience  Individual: All participants in extant study invited  Stratification: none  Neighbourhood definition: 500m crow-fly buffer and participant delimitation | Age, sex, education, housing type, health status, physical functionality | Transport walking (frequency) [not validated]  *FrWalk* | *Perceived:*  Residential density (residential density / urbanisation)  Land use mix – access (access to destinations/services)  Land use mix – diversity (land use mix – destination diversity)  Street connectivity (street connectivity)  Infrastructure for walking/cycling (pedestrian-friendly features)  Aesthetics (greenery and aesthetically pleasing scenery)  Traffic safety (traffic/pedestrian safety)  Crime safety (crime/personal safety)  *Objective:*  Walkability (walkability)  Accessibility (land use mix – destination diversity) | None | Linear regression (clustering not taken into account; transportation frequency apparently normally distributed) | *Main effects with FrWalk:*  Residential density:  b = 1.07 p<.001 (residential density / urbanisation **+**)  Land use mix – access:  b = -0.42 p>.05 (access to destinations/services **0**)  Land use mix – diversity:  b = 0.72 p=.01 (land use mix – destination diversity **+**)  Street connectivity:  b = 0.64 p=.04 (street connectivity **+**)  Infrastructure for walking/cycling:  b = 0.22 p>.05 (pedestrian-friendly features **0**)  Aesthetics:  b = 0.17 p<.001 (greenery and aesthetically pleasing scenery **+**)  Traffic safety:  b = 0.02 p>.05 (traffic/pedestrian safety **0**)  Crime safety:  b = -0.23 p>.05 (crime/personal safety **0**)  *Objective:*  Walkability:  b = 1.05 p>.05 (walkability **0**)  Accessibility:  b = 4.28 p<.001 (land use mix – destination diversity **+**) | Note that there are multiple measures per environmental construct that need to be summed. |
| **26A [66]**  SNQLS  Bracy et al., 2014 | N = 707 (urban)  66+ years; 53% women  21.4% response rate  Community dwellers  Seattle and Baltimore, USA | Cross-sectional  Cluster: purposive  Individuals: random  Stratification: walkability and SES  Neighbourhood definition: 500m street-network buffer and 15-20 min walk from home | Age, sex, education, ethnicity, marital status, months at address, number of people in the household, number of vehicles per adult | Transport walking (min/week) [CHAMPS; validated]  *AmWalk* | *Objective:*  Walkability (walkability)  *Perceived:*  Traffic safety (traffic/pedestrian safety)  Pedestrian safety (traffic/pedestrian safety)  Crime safety (crime/personal safety) | Traffic safety  Pedestrian safety  Crime safety  n average (mean) = 431  n high (+1SD) = 144  n low (-1SD) = 144 | General mixed models accounting for clustering; log transformed transport walking. | *No significant moderating effects*  *Main effects with AmWalk:*  *Objective:*  Walkability:  b ~ 9.00 p <.001 (walkability **+**)  *Perceived:*  Traffic safety:  b = -3.80 p>.05 (traffic/pedestrian safety **0**)  Pedestrian safety:  b = 6.21 p>.05 (traffic/pedestrian safety **0**)  Crime safety:  b = 4.31 p>.05 (crime/personal safety **0**) | Note that there are multiple measures per environmental construct that need to be summed. |
| **27A [32]**  SNQLS  Cain et al., 2014 | N = 367(urban)  66+ years; 51% women  21.4% response rate  Community dwellers  Seattle and Baltimore, USA | Cross-sectional  Cluster: purposive  Individuals: random  Stratification: walkability and SES  Neighbourhood definition: 0.25 mile route from home to nearest pre-determined destination | Age, sex, education, race, physical functioning | Active transport (walking + cycling) (min/week) [CHAMPS; validated]  *AmActTrans* | *Objective (audits):*  Residential mix (residential density / urbanisation)  Shops (shops / commercial)  Restaurant-entertainment (food outlet)  Institutional-service (business / government / institutional / industrial)  Government-service (business / government / institutional / industrial)  Public recreation (park / open space / recreational)  Private recreation (park / open space / recreational)  Parking (other destinations)  Transit stop (public transport)  Negative streetscape characteristics (traffic/pedestrian safety)  Positive aesthetics & social characteristics (greenery and aesthetically pleasing scenery)  Negative aesthetics & social characteristics (litter / vandalism / decay)  Overall crossings/intersections score (traffic/pedestrian safety)  Trees (greenery and aesthetically pleasing scenery)  Building aesthetics/design (greenery and aesthetically pleasing scenery)  Sidewalk (pedestrian-friendly features)  Bike infrastructure (park / open space / recreation)  Buffer (traffic/pedestrian safety)  Negative street-segment characteristics (barriers to walking/cycling)  Positive streetscape characteristics not extracted as they represent a mixture of infrastructure and safety.  Building height – setback item not extracted as they represent a mixture of density and pedestrian-friendly features | None | Multilevel linear regression accounting for clustering  Outcome log-transformed (accounting for positive skewness) | *Main effects with AmActTrans:*  Residential mix:  t = 3.73 p<.001 (residential density / urbanisation **+**)  Shops:  t = 6.14 p<.001 (shops / commercial +)  Restaurant-entertainment:  t = 5.96 p<.001 (food outlet **+**)  Institutional-service:  t = 5.79 p<.001 (business / government / institutional / industrial **+**)  Government-service:  t = 2.66 p<.01 (business / government / institutional / industrial **+**)  Public recreation:  t = -0.41 p>.05 (park / open space / recreational **0**)  Private recreation:  t = 0.68 p>.05 (park / open space / recreational **0**)  Parking:  t = -1.63 p>.05 (other destinations **0**)  Transit stop:  t = 1.32 p>.05 (public transport **0**)  Negative streetscape characteristics:  t = -4.04 p<.001 (traffic/pedestrian safety **+**)  Positive aesthetics & social characteristics:  t = -0.81 p>.05 (greenery and aesthetically pleasing scenery **0**)  Negative aesthetics & social characteristics:  t = 3.38 p<.01 (litter / vandalism / decay **+**)  Overall crossings/intersections score:  t = 4.28 p<.001 (traffic/pedestrian safety **+**)  Trees:  t = 1.83 p>.05 (greenery and aesthetically pleasing scenery **0**)  Building aesthetics/design:  t = 1.65 p>.05 (greenery and aesthetically pleasing scenery **0**)  Sidewalk:  t = 3.53 p<.001 (pedestrian-friendly features **+**)  Bike infrastructure:  t = -0.67 p>.05 (pedestrian-friendly features **0**)  Buffer:  t = 3.76 p<.001 (traffic/pedestrian safety **+**)  Negative street-segment characteristics:  t = -3.53 p<.001 (barriers to walking/cycling **-**) | Note that there are multiple measures per environmental construct that need to be summed. Positive streetscape characteristics not extracted as they represent a mixture of infrastructure and safety.  Building height – setback item not extracted as they represent a mixture of density and pedestrian-friendly features. |
| **28A [65]**  SNQLS  Carlson et al., 2012 | N = 707 (urban)  66+ years; 53% women  21.4% response rate  Community dwellers  Seattle and Baltimore, USA | Cross-sectional  Cluster: purposive  Individuals: random  Stratification: walkability and SES  Neighbourhood definition: 500m street-network buffer and 15-20 min walk from home | Age, sex, education, ethnicity, marital status, months at address, number of people in the household, number of vehicles per adult | Transport walking (min/week) [CHAMPS; validated]  *AmWalk* | *Objective:*  Parks and recreation (parks / open space / recreation)  Walkability not extracted as included in Bracy et al. with more appropriate statistical analyses and moderating effects yielding same-direction associations across values of moderator.  *Perceived:*  Aesthetics (greenery and aesthetically pleasing scenery)  Walking facilities (pedestrian-friendly features) | Social support  Self-efficacy  Barriers  n average (mean) = 431  n high (+1SD) = 144  n low (-1SD) = 144 | General mixed models accounting for clustering but not for positive skewness of transport walking. | *Main effects with AmWalk:*  *Objective:*  Parks and recreation:  b = 9.1 p>.05 (parks / open space / recreation **0**)  *Perceived:*  Aesthetics:  b = 0.5 p>.05 (greenery and aesthetically pleasing scenery **0**)  Walking facilities:  b = 1.1 p>.05 (pedestrian-friendly features **0**)  *Moderating effects:*  Walkability with social support p=.017 (do not count as same direction of effects)  Stronger positive effects in those with higher social support  Walkability with self-efficacy p=.020 (do not count as same direction of effects)  Stronger positive effects in those with higher self-efficacy  Walkability with barriers p=.013 (do not count as same direction of effects)  Stronger positive effects in those with lower levels of barriers | Walkability not extracted as included in Bracy et al. with more appropriate statistical analyses and moderating effects yielding same-direction associations across values of moderators. |
| **29A [22]**  SNQLS  Ding et al. 2014 | N = 880 (urban)  66+ years; 56% women  24.5% response rate  Community and retirement village dwellers  Seattle and Baltimore, USA | Cross-sectional with two assessments  Cluster: purposive  Individuals: random  Stratification: walkability and SES  Neighbourhood definition: 500m street-network buffer and 15-20 min walk from home | Age, sex, education, ethnicity, study site, marital status, number of people in household, living situation, length of time in current address, medical conditions, mobility impairment | Transport walking (Yes/No) [CHAMPS; validated]  *Walk(YN)* | *Objective:*  Walkability (walkability)  Parks and recreational facilities (categorized) (parks / open space / recreation)  *Perceived:*  Residential density (residential density / urbanisation)  Land use mix – access (access to destinations/services)  Land use mix – diversity (land use mix – destination diversity)  Street connectivity (street connectivity)  Walking or cycling infrastructures (pedestrian-friendly features)  Neighbourhood aesthetics (greenery and aesthetically pleasing scenery)  Traffic safety (traffic/pedestrian safety)  Pedestrian safety structures (traffic/pedestrian safety)  Transit access (public transport)  Personal safety (crime/personal safety) | Driving status  n (driving) = 726  n (non-driving) = 154 | Generalized mixed linear regression models with binomial variance and logit link functions (accounting for clustering at the census block level) | *No significant moderating effects*  *Main effect with Walk(YN):*  *Objective:*  Walkability:  ORs indicative of positive association with p<.001 (walkability **+**)  Parks and recreational facilities:  ORs and trends indicative of positive association one p<.001 (parks / open space / recreation **+**)  *Perceived:*  Residential density:  ORs indicating of positive association p<.05 (residential density / urbanisation **+**)  Land use mix – access:  ORs indicating of positive association p<.01 (access to destinations/services **+**)  Land use mix – diversity:  ORs indicating of positive association p<.001 (land use mix – destination diversity **+**)  Street connectivity:  ORs and trends indicative of positive association one p<.01 (street connectivity **+**)  Walking or cycling infrastructures:  ORs indicating of positive association p<.001 (pedestrian-friendly features **+**)  Neighbourhood aesthetics:  ORs indicating of positive association p<.05 (greenery and aesthetically pleasing scenery **+**)  Traffic safety:  ORs p>.05 (traffic/pedestrian safety **0**)  Pedestrian safety structures:  ORs indicating of positive association p<.05 (traffic/pedestrian safety **+**)  Transit access:  ORs indicating of positive association p<.01 (public transport **+**)  Personal safety:  ORs p>.05 (crime/personal safety **0**) | Only SNQLS paper to examine transport walking as a dichotomous measure (Yes/No). Report all findings.  Note that there are multiple measures per environmental construct that need to be summed |
| **30A [34]**  SNQLS  King et al., 2011 | N = 719 (urban)  66+ years; 53% women  21.4% response rate  Community dwellers  Seattle and Baltimore, USA | Cross-sectional with two assessments  Cluster: purposive  Individuals: random  Stratification: walkability and SES  Neighbourhood definition: Census block group | Age, sex, education, ethnicity, number of motor vehicles in household, study site, marital status, number of people in household, length of time in current address | Active transport (walking + cycling) [CHAMPS; validated]  *AmActTrans* | *Objective:*  Walkability – dichotomised (walkability) | Mobility impairment  Area-level household income | General mixed models accounting for repeated measures and clustering but not for positively skewed outcome. | *Main effect with AmActTrans:*  F statistic for main effect = 32.82 p<.001 (walkability **+**)  *Moderating effects:*  Walkability with mobility impairment p<.001 (do not count as same direction of effects)  Stronger positive effects in least mobility impaired | Included because it is the only SNQLS paper to assess active transport (walking + cycling). Otherwise, it would have been excluded as continuous walkability measure and Walk(YN) reported in Ding et al. |
| **31A [63]**  SNQLS  Shigematsu et al., 2009 | N = 360 (urban)  66+ years; 51% women  23% response rate  Community dwellers  Seattle and Baltimore, USA | Cross-sectional  Cluster: purposive  Individuals: random  Stratification: walkability and SES  Neighbourhood definition: 15-20 min walk from home | Sex, BMI, education, income, driver’s licence | Transport walking (min/week) [CHAMPS; validated]  *AmWalk* | *Perceived:*  Residential density (residential density / urbanisation)  Land use mix – access (access to destinations/services)  Land use mix – diversity (land use mix – destination diversity)  Street connectivity (street connectivity)  Recreational facilities near home (parks / open space / recreation)  Park near home (parks / open space / recreation)  Other NEWS attributes not extracted as reported in Carlson et al. and Bracy et al. on the whole SNQLS sample, while this study reports data from a portion of the sample. | Age  n (66-75 years) = 201  n (76+ years) = 159 | Partial correlations (not accounting for clustering or non-normality of outcome). No formal moderation analyses. | *Main effects with AmWalk:*  Residential density:  Partial r positive in one group p<.05; similar in other group (residential density / urbanisation **+**)  Land use mix – access:  Partial r positive p<.05 (access to destinations/services **+**)  Land use mix – diversity:  Partial r positive p<.05 (land use mix – destination diversity **+**)  Street connectivity:  Partial r p>.05 (street connectivity **0**)  Recreational facilities near home:  Partial r positive p<.05 (parks / open space / recreation **+**)  *Moderating effects:*  Park near home:  66-75 years: partial r = 0.02 p>.05 (parks / open space / recreation **0 * 0.56**)  76+ years: partial r = 0.30 p<.05 (parks / open space / recreation **+ * 0.44**) | Some moderating effects with factional weights. Note that there are multiple measures per environmental construct that need to be summed. Only 6 NEWS attributes extracted; other reported in Carlson et al. and Bracy et al. on the whole SNQLS sample, while this stud reports data from a portion of the sample. |
| **32A [67]**  SMARTRAQ  Frank et al., 2010 | N = 1970 (urban)  65+ years; 56% women  30% response rate  Community dwellers  Atlanta, USA | Cross-sectional  Cluster: all counties  Individual: systematic  Stratification: household size and income, residential density  Neighbourhood definition: 1km street-network buffer around home | Age, living arrangement, household income, number of cars in households, ethnicity, education, sex | Transport walking – frequency (trips/2 days YN) [travel diary; validated]  *Walk(YN)* | *Objective:*  Walkability (walkability) | None | Multilevel logistic regression accounting for clustering (quantiles of continuous exposure) | *Main effects with Walk(YN):*  Walkability:  OR = 2.02 p<.05 for high walkability vs low walkability (walkability **+**) |  |
| **33A [55]**  Walk the Talk  Chudyk et al., 2015 | N = 150 (urban)  Median = 74 years; 66% women  8% response rate  Community dwellers  Vancouver, Canada | Cross-sectional  Households: stratified random  Individual: not reported  Stratification: walkability  Neighbourhood definition: 1.5 mile from home | Age, sex, living arrangement, enjoyment of walking, vehicle in household, comorbidities | Transport walking (trips/day) (travel diary; validated]  *FrWalk* | *Objective:*  Street Smart Walk Score (walkability) | None | Negative binomial regression with robust standard errors accounting for clustering at the city level | *Main effects with FrWalk*:  Street Smart Walk Score:  exp (b) = 1.20 p<.001 (walkability **+**) |  |
| **34A [62]**  None  Barnes et al, in press | N = 780 (urban)  65+ years; 52% women  74% response rate  Community dwellers  Metropolitan area of British Colombia, Canada | Cross-sectional  Cluster: representative sample  Individuals: random sampling  Stratification: none  Neighbourhood definition: postal code | Sex, age, country of birth, education, valid driver’s license | Walking for transport (Yes/No) [extracted from travel diaries] [validated]  *Walk(YN)* | *Objective:*  Street Smart Walk Score (walkability)  Transit Score (public transport) | Age  n (65-74) = 422  n (75+) = 358 | Logistic regression with survey weights and sensitivity analysis to assess clustering; formal testing for moderators | No moderating effects.  *Main effects with Walk(YN)*:  Walk Score: OR = 1.34; 95% CI: 1.23, 1.47 (Walkability **+**)  Transit Score: OR = 1.28; 95% CI: 1.17, 1.40 (Public transport **+**) | None |
| **35A [98]**  None  Garrard, 2013 | N = 1128 (not reported)  Mean age likely 65+ years; 74%v women  Response rate not quantifiable  Community dwellers  Victoria, Australia | Cross-sectional  Cluster: regions in Victoria  Individual: convenience  Stratification: none  Neighbourhood definition: postcode | None | Transport walking (min/week) dichotomised (60+ YN) [survey; not validated]  *Walk(60+ YN)* | *Objective:*  Regions in Victoria (residential density / urbanisation) | None | Chi-square test | *Main effects with Walk(60+ YN):*  Chi-square test p<.05 indicating higher prevalence of walking in more urbanised areas (residential density / urbanisation **+**) | Grey literature |
| **36A [35]**  None  Inoue et al., 2011 | N = 1921 (urban and rural)  65+ years; 49% women  73% response rate  Community dwellers  Oyama, Bunkyo and Fuchu, Japan | Cross-sectional  Cluster: random  Individual: random  Stratification: urban, suburban and rural  Neighbourhood definition: 10-15 min walk from home | Age, sex, city of residence, employment status, education, BMI, self-rated health | Transportation walking (60+ min/wk YN) [modification of previously validated questionnaire]  *Walk(60+YN)* | *Perceived:*  Residential density (residential density / urbanisation)  Access to shops (shops/commercial)  Access to public transport (public transport)  Sidewalks (pedestrian-friendly features)  Bicycle lanes (pedestrian-friendly features)  Access to exercise facilities (park / open space / recreation)  Crime safety (crime/personal safety)  Traffic safety (traffic / pedestrian safety)  Aesthetics (greenery and aesthetically pleasing scenery) | Sex  n (men) = 977  n (women) = 944 | Multilevel logistic regression accounting for clustering | *Main effects with Walk(60+YN):*  Residential density:  OR = 1.03 p>.05 (residential density / urbanisation **0**)  Public transport:  OR = 1.03 p>.05 (public transport **0**)  Sidewalks:  OR = 1.19 p>.05 (pedestrian-friendly features **0**)  *Moderating effects:*  Access to shops:  Men: OR = 0.95 p>.05 (shops/commercial **0*0.51**)  Women: OR = 1.57; p=.004 (shops/commercial **+*0.49**)  Bicycle lanes:  Men: OR = 1.41 p=.019 (pedestrian-friendly features **+*0.51**)  Women: OR = 1.14; p>.05 (pedestrian-friendly features **0*0.49**)  Access to exercise facilities:  Men: OR = 1.16 p>.05 (park / open space / recreation **0*0.51**)  Women: OR = 1.39; p=.033 (park / open space / recreation **+*0.49**)  Crime safety:  Men: OR = 0.69 p=.017 (crime/personal safety **-*0.51**)  Women: OR = 1.07; p>.05 (crime/personal safety **0*0.49**)  Traffic safety:  Men: OR = 0.71 p=.017 (traffic / pedestrian safety **-*0.51**)  Women: OR = 1.15; p>.05 (traffic / pedestrian safety **0*0.49**)  Aesthetics:  Men: OR = 1.33 p=.047 (greenery and aesthetically pleasing scenery **+*0.51**)  Women: OR = 1.28; p>.05 (greenery and aesthetically pleasing scenery **0*0.49**) | Note that there are multiple measures per environmental construct that need to be summed. Fractional weights needed as there moderating effect of sex. |
| **37A [47]**  None  Kolbe-Alexander et al., 2015 | N = 44 (urban)  Mean age = 65; 78% women  Response rate not reported  Community dwellers  Cape Town, South Africa | Cross-sectional  Cluster: purposive  Individual: convenience  Stratification: area-level SES  Neighbourhood definition: participant delimitation | None | Active transport (min/wk) [GPAQ; validated]  *AmActTrans* | *Perceived:*  Residential density (residential density / urbanisation)  Land use mix – access (access to destinations/services)  Land use mix – diversity (land use mix – destination diversity)  Street connectivity (street connectivity)  Walking or cycling infrastructure (pedestrian-friendly features)  Aesthetics (greenery and aesthetically pleasing scenery)  Safety from traffic (traffic/pedestrian safety)  Safety from crime(crime/personal safety) | Area-level SES  n (high SES) = 20  n (low SES) = 24 | Spearman correlation coefficient | *Main effects with AmActTrans:*  Residential density:  r p>.05 (residential density / urbanisation **0**)  Land use mix – access:  r p>.05 (access to destinations/services **0**)  Land use mix – diversity:  r p>.05 (land use mix – destination diversity **0**)  Street connectivity:  r p>.05 (street connectivity **0**)  Aesthetics:  r p>.05 (greenery and aesthetically pleasing scenery **0**)  Safety from traffic:  r = -0.37 p=.01 (traffic/pedestrian safety **-**)  Safety from crime:  r p>.05 (crime/personal safety **0**)  *Moderating effects:*  Walking or cycling infrastructure:  Low SES: r p>.05 (pedestrian-friendly features **0*0.55**)  High SES: r=0.55 p=.009 (pedestrian-friendly features **+*0.45**) | Fractional weights needed as there moderating effect of area-level SES. |
| **38A [48]**  None  Maisel, 2016 | N = 121 (urban, suburban, rural)  65+ years; 74% women  Response rate not reported  Community dwellers  Erie County NY, USA | Cross-sectional  Cluster: purposive  Individual: convenience  Stratification: area-level SES / urbanisation  Neighbourhood definition: participant delimitation | Age, sex, household income | Transport walking (YN) [IPAQ-L; validated]  *Walk(YN)*  *Note: Total weekly minutes of transport walking were also assessed but the analyses were not adjusted for socio-demographics. Hence, they were not included in this review.* | *Perceived:*  Residential density (residential density / urbanisation)  Land use mix – access (access to destinations/services)  Land use mix – diversity (land use mix – destination diversity)  Street connectivity (street connectivity)  Walking or cycling infrastructure (pedestrian-friendly features)  Aesthetics (greenery and aesthetically pleasing scenery)  Safety from traffic (traffic/pedestrian safety)  Safety from crime(crime/personal safety) | Urbanisation  n (urban) = 32  n (suburban) = 50  n (rural) = 39 | Logistic regression | *Main effects with Walk(YN):*  Residential density:  OR p>.05 (residential density / urbanisation **0**)  Land use mix – access:  OR p>.05 (access to destinations/services **0**)  Land use mix – diversity:  OR p>.05 (land use mix – destination diversity **0**)  Street connectivity:  OR p>.05 (street connectivity **0**)  Aesthetics:  OR p>.05 (greenery and aesthetically pleasing scenery **0**)  Safety from traffic:  OR p>.05 (traffic/pedestrian safety **0**)  Safety from crime:  OR p>.05 (crime/personal safety **0**)  Walking or cycling infrastructure:  OR p>.05 (pedestrian-friendly features **0**)  No moderating effects of urbanisation. |  |
| **39A [56]**  None  Mitchell, 2012 | N = 140 (urban)  65+ years; 69% women  Response rate not reported  Community dwellers  Philadelphia, USA | Cross-sectional  Cluster: community sites  Individual: convenience  Stratification: none  Neighbourhood definition: 4 block radius (~20 min walk from home) | Age, sex, BMI, race, length of residence, health variables, drives a car | Transport walking (hr/wk) [CHAMPS; validated]  *AmWalk* | *Perceived:*  Residential density (residential density / urbanization)  Land use mix – diversity (land use mix – destination diversity)  Land use mix - access to services (access to destination / services)  Street connectivity (street connectivity)  Infrastructure for cycling/walking (pedestrian and cycling infrastructure)  Aesthetics (aesthetics and cleanliness)  Traffic (traffic/pedestrian safety)  Crime (crime/personal safety) | None | Linear regression not accounting for clustering (community sites) and positively skewed outcome | *Main effects with AmWalk:*  Residential density:  b p>.05 (residential density / urbanization **0**)  Land use mix – diversity:  b p>.05 (land use mix – destination diversity **0**)  Land use mix - access to services:  b = 45.05 p=.005 (access to destination / services **+**)  Street connectivity:  b p>.05 (street connectivity **0**)  Infrastructure for cycling/walking:  b p>.05 (pedestrian-friendly features **0**)  Aesthetics:  b p>.05 (aesthetics and cleanliness **0**)  Traffic:  b p>.05 (traffic/pedestrian safety **0**)  Crime:  b p>.05 (crime/personal safety **0**) | Grey literature – PhD thesis |
| **40A [49]**  None  Patterson et al., 2004 | N = 372 (urban and suburban)  70+ years; 100% women  40% response rate  Community dwellers  Portland OR, USA | Cross-sectional  Cluster: purposive  Individual: systematic  Stratification: walkability  Neighbourhood definition: 0.25 mile buffer around the home | Income, health, driving ability, years lived in the house (single sex sample) | Transport walking (number of services accessed by walking) [not validated]  *FrWalk* | *Objective:*  New Urbanism Index (walkability) | None | Linear regression (clustering not taken into account; distributional characteristics of outcome not reported] | *Main effects with FrWalk:*  New Urbanism Index:  r^2^ = 0.23 p<.001 (walkability **+**) |  |
| **41A [96]**  None  Pelclova et al., 2012 | N = 456 (not reported)  Mean age ~ 65 years; 88% women  Response rate not reported  Community dwellers  Czech Republic, Poland and Slovakia | Cross-sectional  Individual: all eligible participants enrolled in University of Third Age invited  Stratification: none  Neighbourhood definition: participant delimitation | Age, BMI, dog ownership, bicycle ownership, city size measured but not entered as co-variates | Transportation walking (150+ min/wk YN)  [IPAQ-L; validated]  *Walk(150+YN)* | *Perceived:*  Residential density (residential density / urbanisation)  Land use mix – access (access to destinations/services)  Land use mix – diversity (land use mix – destination diversity)  Street connectivity (street connectivity)  Infrastructure for walking/cycling (pedestrian-friendly features)  Neighbourhood aesthetics (greenery and aesthetically pleasing scenery)  Traffic and crime safety (safety and traffic) | None | Logistic regression (study site not accounted for; all environmental variables adjusted for other environmental variables) | *Main effects with Walk(150+YN):*  Residential density:  OR = 1.87 p<.05 (residential density / urbanisation **+**)  Land use mix – access:  OR = 1.29 p>.05 (access to destinations/services **0**)  Land use mix – diversity:  OR = 1.02 p>.05 (land use mix – destination diversity **0**)  Street connectivity:  OR = 1.80 p>.05 (street connectivity **0**)  Infrastructure for walking/cycling:  OR = 1.54 p>.05 (pedestrian-friendly features **0**)  Neighbourhood Aesthetics:  OR = 1.32 p>.05 (greenery and aesthetically pleasing scenery **0**)  Traffic and crime safety:  OR = 0.74 p>.05 (safety and traffic **0**) |  |
| **42A [50]**  None  Sugiyama & Thompson, 2008 | N = 268 (urban and rural)  65+ years; 61% women  Various response rate in subsamples (9%; 47%, not stated)  Community dwellers and sheltered housing  Great Britain | Cross-sectional  Cluster: purposive  Individual: various strategies  Stratification: social deprivation, urbanisation, type of employment  Neighbourhood definition: local councils | Age, sex, education, former occupation | Transportation walking (60+ min YN) [not validated]  *Walk(60+ YN)* | *Perceived:*  Neighbourhood open space (NOS) pleasantness (greenery and aesthetically-pleasing scenery)  Good paths to NOS (pedestrian-friendly features)  Safety (crime/personal safety)  Good facilities (access to services/destinations)  Nuisances (litter / vandalism / decay)  Water feature (greenery and aesthetically-pleasing scenery) | None | Logistic regression models (clustering not accounted for) | *Main effects with Walk(60+ YN):*  Neighbourhood open space (NOS) pleasantness:  OR p>.05 (greenery and aesthetically-pleasing scenery **0**)  Good paths to NOS:  OR = 1.38 p<.05 (pedestrian-friendly features **+**)  Safety:  OR p>.05 (crime/personal safety **0**)  Good facilities:  OR = 1.34 p<.05 (access to services/destinations **+**)  Nuisances:  OR p>.05 (litter / vandalism / decay **0**)  Water feature:  OR p>.05 (greenery and aesthetically-pleasing scenery **0**) | Note that there are multiple measures per environmental construct that need to be summed. |
