# Supplementary material for: The neighbourhood physical environment and active travel in older adults: a systematic review and meta-analysis
Source: Int J Behav Nutr Phys Act. 2017 Feb 6;14:15. doi: 10.1186/s12966-017-0471-5 (PMC5294838; doi:10.1186/s12966-017-0471-5)
Supplement: Additional file 3: — Supplementary analytical example – computation of p-values for associations of food outlets with all active travel outcomes in older adults. (DOCX 26 kb) [file 12966_2017_471_MOESM3_ESM.docx]

**Supplementary analytical example – computation of p-values for associations of food outlets with all active travel outcomes in older adults**

The aim of this section is to provide an example of how p-values adjusted for both sample size and article quality were computed. We do this for the associations reported on access to, or availability of, food outlets and all active travel outcomes.

Supplementary Table 1 indicates that four articles examined the associations between access to, or availability of, food outlets and active travel. The articles’ sample sizes and sample size scores, quality scores and findings are summarised in the table below. The following weights were assigned for sample size: 0.25 for a sample of ≤100 participants; 0.50 for 101-300 participants; 1.00 for 301-500 participants; 1.25 for 501-1000 participants; 1.50 for 1001-2500 participants; and 1.75 for >2500 participants. A quality score was derived from Supplementary Table 2. Positive associations were assigned a z-value of 1.96 (just significant at a p-level of 0.05), negative associations were given a z-value of -1.96 and statistically non-significant associations a z-value of 0. In articles that found significant moderators of environment-AT associations and had inconsistent associations across values of the moderator, associations at each examined value of the moderator were assigned fractional weights corresponding to the (approximate) proportion of the total sample represented by the subgroup of participants. For continuous moderators (as in this example), associations computed at the average value of the moderator were assigned a weight of 0.60, while those at 1 standard deviation (SD) below and above the mean were each assigned a weight of 0.20 (the total sum of the weights is 1). The logic behind this is that, under the Normal distribution, the proportions of values 1SD above and below the mean are ~ 20% (factoring some uncertainty around the value of the moderator at +1SD and -1SD). If an association was moderated by multiple factors (two factors in the example below), weights were assigned following the logic described above but in such a fashion that the sum of the weights across all examined values of all the significant moderators was 1. This was done by dividing the weights 0.60, 0.20 and 0.20 by the number of significant moderators. In this case, we get 0.30, 0.10 and 0.10 as we had two significant moderators of specific food outlet measures and active travel.

***Supplementary Analytical Example - Table 1 - Article characteristics and findings***

| **Article number [reference # in review]**  Article  (Study) | Sample size score  (sample size) | Quality score | Descriptive findings | # finding (j) | Coding of findings - assigned z-values (fractional weights) |
| --- | --- | --- | --- | --- | --- |
| **27A [32]**  Cain et al. 2016  (SNQLS study) | 1.25  (367) | 5.00 | Positive association between restaurant – entertainment and walking + cycling | 1 | 1.96 (1.00) |
| **16A [51]**  Cerin et al., 2013  (HK Elderly 1; IJBNPA) | 1.00  (484) | 6.00 | Statistically non-significant associations:  Food and grocery store – prevalence and total walking  Food and grocery store – diversity and total walking  Food and grocery store – diversity and within-neighbourhood walking  Restaurant – prevalence and total walking  Restaurant – diversity and total walking  Restaurant – diversity and within-neighbourhood walking  Food and grocery store – prevalence and within-neighbourhood walking at high levels of path obstructions (1SD above the mean)  Food and grocery store – prevalence and within-neighbourhood walking at high levels of sloping streets (1SD above the mean)  Positive associations:  Restaurant – prevalence and within-neighbourhood walking  Food and grocery store – prevalence and within-neighbourhood walking at low levels of path obstructions (1SD below the mean)  Food and grocery store – prevalence and within-neighbourhood walking at low levels of sloping streets (1SD below the mean)  Food and grocery store – prevalence and within-neighbourhood walking at average levels of path obstructions  Food and grocery store – prevalence and within-neighbourhood walking at average levels of sloping streets | 2  3  4  5  6  7  8  9  10  11  12  13  14 | 0.00 (1.00)  0.00 (1.00)  0.00 (1.00)  0.00 (1.00)  0.00 (1.00)  0.00 (1.00)  0.00 (0.10)  0.00 (0.10)  1.96 (1.00)  1.96 (0.10)  1.96 (0.10)  1.96 (0.30)  1.96 (0.30) |
| **21A [70]**  Moniruzzaman et al., 2015  (Montreal’s Household Travel Survey) | 1.75  (likely >15,000) | 3.67 | Statistically non-significant association between distance to nearest grocery store and total walking | 15 | 0.00 (1.00) |
| **19A [92]**  Procter-Gray et al., 2015  (MOBILIZE) | 1.25  (745) | 3.67 | Negative associations between distance to grocery/convenience store and total walking | 16 | 1.96 (1.00) |

The table above indicates that we have 7.2 non-significant associations and 3.8 positive associations, a distribution of findings that is typically ‘subjectively’ described as not supportive of a positive association. To obtain the p-value of the distribution of findings observed above, we used the following formula $Weighted Z= \frac{\sum{weight}_{j}z_{j}}{\sqrt{\sum{weight}_{j}^{2}}}$ , where ‘j’ stands for finding ‘j’, z_j_ correspond to the z-value assigned to finding ‘j’ (from the table above), and ‘weight’ for the ‘j’ finding corresponds to the sum of the sample size score and quality score of a specific article multiplied by the fractional weight of finding ‘j’. The computations are presented in Table 2 below.

***Supplementary Analytical Example - Table 2 – p-value computation***

| Article (Study) | Finding (j) | Weight_j_ | Weight_j_^2^ | z_j_ | Weight_j_ * z_j_ |
| --- | --- | --- | --- | --- | --- |
| Cain et al. 2016 (SNQLS study) | 1 | 6.25 | 39.0625 | 1.96 | 12.25 |
| Cerin et al., 2015 (HK Elderly 1) | 2 | 7.00 | 49.00 | 0.00 | 0.00 |
|  | 3 | 7.00 | 49.00 | 0.00 | 0.00 |
|  | 4 | 7.00 | 49.00 | 0.00 | 0.00 |
|  | 5 | 7.00 | 49.00 | 0.00 | 0.00 |
|  | 6 | 7.00 | 49.00 | 0.00 | 0.00 |
|  | 7 | 7.00 | 49.00 | 0.00 | 0.00 |
|  | 8 | 0.70 | 0.49 | 0.00 | 0.00 |
|  | 9 | 0.70 | 0.49 | 0.00 | 0.00 |
|  | 10 | 7.00 | 49.00 | 1.96 | 13.72 |
|  | 11 | 0.70 | 0.49 | 1.96 | 1.372 |
|  | 12 | 0.70 | 0.49 | 1.96 | 1.372 |
|  | 13 | 2.10 | 4.41 | 1.96 | 4.116 |
|  | 14 | 2.10 | 4.41 | 1.96 | 4.116 |
| Moniruzzaman et al., 2015 (Montreal’s Household Travel Survey) | 15 | 5.42 | 29.3764 | 0.00 | 0.00 |
| Procter-Gray et al., 2015 (MOBILIZE) | 16 | 4.92 | 24.2064 | 1.96 | 9.6432 |
| Total sum (∑) | - | - | 446.4253 | - | **46.5812** |
| Square root | - | - | **21.1288** | - | - |

By replacing the weighted sum of z-values (46.5812) and the square root of the sum of squared weights (21.1288) into the Weighted Z formula above we get:

$Weighted Z= \frac{46.5812}{21.1288}=2.205$, which is associated with a two-tailed probability value of 0.027. In contrast to the typical ‘subjective’ conclusions derived from the an examination of the number of significant positive and nil findings described, this quasi-meta-analytical approach suggests that there is support for a positive relationship between food outlets in the neighbourhood and active travel in older adults. In fact, a distribution of findings supportive of no association would be likely to have an approximately balanced number of positive and negative associations and mostly nil findings.
