# Supplementary material for: The neighbourhood physical environment and active travel in older adults: a systematic review and meta-analysis
Source: Int J Behav Nutr Phys Act. 2017 Feb 6;14:15. doi: 10.1186/s12966-017-0471-5 (PMC5294838; doi:10.1186/s12966-017-0471-5)
Supplement: Additional file 4: Table S2. — Article quality assessment. (DOCX 28 kb) [file 12966_2017_471_MOESM4_ESM.docx]

**Supplementary Table 2 - Article quality assessment**

| Article number, study name and authors | Recruitment stratified by key environmental attributes  [weight 1] | Response rate ≥60% or sample shown to be representative of the population  [weight 1] | Outcome measures shown to be valid or commonly-used  [weight 1] | Adjustment for socio-demographic covariates (at least age, sex, education or similar)  [weight 1] | Adjustment for self-selection  [weight 1] | Appropriate analytical approach – accounting for clustering (if needed)  [weight 1/3] | Appropriate analytical approach – accounting for distributional assumptions  [weight 1/3] | Appropriate analytical approach –analyses conducted and presented correctly (e.g., formal testing of moderators; presentation of point estimates and p-values, 95% CIs)  [weight 1/3] | Did not (inappropriately) categorise continuous environmental exposure  [weight 1] | Total quality score (out of 7) |
| --- | --- | --- | --- | --- | --- | --- | --- | --- | --- | --- |
| **1A.** Active Living Study  Boruff et al., 2012 | Y | N | Y | Y | N | Y | Y | Y | Y | 5 |
| **2A.** Active Living Study  Nathan et al., 2014 (AJAG) | Y | N | Y | Y | N | Y | Y | Y | Y | 5 |
| **3A.** Active Living Study  Nathan et al., 2014 (Environment & Behavior) | Y | N | Y | Y | Y | Y | Y | Y | Y | 6 |
| **4A.** Active Living Study  Nathan et al., 2014 (JAPA) | Y | N | Y | Y | Y | Y | Y | Y | Y | 6 |
| **5A.** ALECS  Barnett et al., 2016 | Y | Y | Y | Y | N | Y | Y | Y | Y | 6 |
| **6A.** BCC initiative – Rode Island Trial  King et al. 2006 | N | N | Y | Y | N | Y | N | Y | Y | 3.67 |
| **7A.** Belgian Aging Studies  Van Cauwenberg et al., 2012 | Y | Y | N | Y | N | Y | Y | Y | Y | 5 |
| **8A.** BEPAS Seniors  Van Cauwenberg et al., 2016 | Y | N | Y | Y | Y | Y | Y | Y | Y | 6 |
| **9A.** BEPAS Seniors  Van Holle et al., 2014 | Y | N | Y | Y | N | Y | Y | Y | Y | 5 |
| **10A.** BEPAS Seniors  Van Holle et al., 2016 | Y | N | Y | Y | N | Y | Y | Y | Y | 5 |
| **11A.** CNDS  Mendes de Leon, 2019 | N | Y | N (previously used but validity not reported) | Y | N | Y | Y | Y | Y | 4 |
| **12A.** ELANE  Etman et al., 2014 | N | N | Y | Y | N | Y | Y | Y | Y | 4 |
| **13A.** EpiFloripa Elderly  Corseuil et al., 2011 | Y | Y | Y | Y | N | Y | Y | Y | Y | 6 |
| **14A.** EpiFloripa Elderly  Corseuil et al., 2016 (JAH) | Y | Y | Y | Y | N | Y | Y | Y | Y | 6 |
| **15A.** EpiFloripa Elderly  Corseuil et al., 2016 (JPAH) | Y | Y | Y | Y | N | Y | Y | Y | N (dichotomization and use of tertiles) | 5 |
| **16A.** HK elderly 1  Cerin et al., 2013 (HKMJ) | Y | Y | Y | Y | N | Y | Y | Y | Y | 6 |
| **17A.** HK elderly 1  Cerin et al., 2013 (IJBNPA) | Y | Y | Y | Y | N | Y | Y | Y | Y | 6 |
| **18A.** HK elderly 1  Cerin et al., 2014 (PHN) | Y | Y | Y | Y | N | Y | Y | Y | Y | 6 |
| **20A.** MOBILIZE Boston Study  Procter-Gray et al., 2015 | N | Y | N | Y | N | N (not adjusted for clustering at the community level) | Y | Y | Y | 3.67 |
| **21A.** Montreal’s Household Travel Survey  Moniruzzaman et al., 2013 | N | N | Y | Y | N | N (not reported) | Y | Y | Y | 3.67 |
| **22A.** Montreal’s Household Travel Survey  Moniruzzaman et al., 2015 | N | N | Y | Y | N | N | Y | Y | Y | 3.67 |
| **23A.** NASH  King, 2008 | Y | N | Y | Y | N | Y | Y | Y | Y | 5 |
| **24A.** Project OPAL  Davis et al. 2011 | Y | N (only reported representativeness by age, sex and BMI) | N (validity not discussed/referenced) | N | N | N (not accounted for clustering at the clinic level) | Y | Y | Y | 2.67 |
| **25A.** SCAMOB Project  Tsai et al., 2013 | N | N | N | Y | N | Y | Y | Y | N | 2 |
| **26A.** Singapore Longitudinal Aging Study  Nyut et al., 2015 | N | N | N | Y | N | N (not reported) | Y | Y | N (categorized continuous objective environmental measures) | 1.67 |
| **27A.** SNQLS  Bracy et al., 2014 | Y | N (🡩 % Caucasians; higher education) | Y | Y | N | Y | Y | Y | Y | 5 |
| **28A.** SNQLS  Cain et al., 2014 | Y | N | Y | Y | N | Y (assuming data is aggregated by subject) | Y | Y (point estimates missing; justification provided) | Y | 5 |
| **29A.** SNQLS  Carlson et al., 2012 | Y | N (🡩 % Caucasians; higher education) | Y | Y | N | Y | N | N | Y | 4.33 |
| **30A.** SNQLS  Ding et al. 2014 | Y | N (🡩 % Caucasians; higher education) | Y | Y | N | Y | Y | Y | N (categorization of # of recreational facilities without justification) | 4 |
| SNQLS  King et al., 2011 | Y | N (🡩 % Caucasians; higher education) | Y | Y | N | Y | N (transport walking positively skewed; used Gaussian models) | N (moderation analyses did not provide point estimates and p-values of slopes) | N (dichotomised continuous walkability index) | 3.33 |
| SNQLS  Shigematsu et al., 2009 | Y | N | Y | Y | N | N | N | N | Y | 4 |
| SMARTRAQ  Frank et al., 2010 | Y | N | Y | Y | N | Y | Y | Y | N | 4 |
| Walk the Talk  Chudyk et al., 2015 | Y | N | Y | Y | Y (enjoying walking) | Y | Y | Y | Y | 6 |
| None  Barnes et al, in press | N | Y | Y | Y | N | Y | Y | Y | Y | 5 |
| None  Garrard, 2013 | N | N | N | N | N | Y | Y | Y | Y | 2 |
| None  Inoue et al., 2011 | Y | Y | N (non-validated modification of extant questionnaire) | Y | N | Y | Y | N (did not formally test for moderating effects of sex) | N (dichotomization of 4-point scales) | 3.67 |
| None  Kolbe-Alexander et al., 2015 | Y | N | Y | N | N | N | Y | N | Y | 3.33 |
| None  Maisel, 2016 | Y | N | Y | Y | N | N | Y | N | N | 3.33 |
| None  Mitchell, 2012 | N | N | Y | Y | N | N | N | Y | y | 3.33 |
| None  Patterson et al., 2004 | Y | N | N | Y | N | N | N | N (only % variance explained reported) | Y | 3 |
| None  Pelclova et al., 2012 | N | N | Y | N | N | N (analyses not adjusted for study site - country) | Y | Y | N | 1.67 |
| Sugiyama & Thompson, 2008 | Y | N | N | Y | N | N | Y | Y | Y | 3.67 |
