# Supplementary material for: The neighbourhood physical environment and active travel in older adults: a systematic review and meta-analysis
Source: Int J Behav Nutr Phys Act. 2017 Feb 6;14:15. doi: 10.1186/s12966-017-0471-5 (PMC5294838; doi:10.1186/s12966-017-0471-5)
Supplement: Additional file 5: Table S3. — Summary table of meta-analytic results of significance of associations of neighbourhood built environmental correlates of active travel outcomes in older adults by type of adjustment for article characteristics. (DOCX 35 kb) [file 12966_2017_471_MOESM5_ESM.docx]

**Supplementary Table 3. Summary table of meta-analytic-results of significance of associations of neighbourhood built environmental correlates of active travel outcomes in older adults by type of adjustment for article characteristics**

| Environmental attributes | Total walking | | | | |  | Within-neighbourhood walking | | | | |  | Walking + cycling | | | | |  | Cycling | | | | |  | All active travel | | | | |
| --- | --- | --- | --- | --- | --- | --- | --- | --- | --- | --- | --- | --- | --- | --- | --- | --- | --- | --- | --- | --- | --- | --- | --- | --- | --- | --- | --- | --- | --- |
|  | p_a_ | p_sa_ | p_q_ | p_u_ | Diff |  | p_a_ | p_sa_ | p_q_ | p_u_ | Diff |  | p_a_ | p_sa_ | p_q_ | p_u_ | Diff |  | p_a_ | p_sa_ | p_q_ | p_u_ | Diff |  | p_a_ | p_sa_ | p_q_ | p_u_ | Diff |
| *Residential density / urbanisation* | <.001 | <.001 | <.001 | <.001 | No |  | .319 | .279 | .327 | .327 | No |  | .089 | .055 | .103 | .166 | No |  | .050 | .050 | .050 | .050 | No |  | .002 | <.001 | .002 | <.001 | No |
| *Walkability* | <.001 | <.001 | <.001 | <.001 | No |  | - | - | - | - | - |  | .050 | .050 | .050 | .050 | No |  | 1.00 | 1.00 | 1.00 | 1.00 | No |  | <.001 | <.001 | <.001 | <.001 | No |
| *Street connectivity* | .014 | .005 | .021 | .011 | No |  | .046 | .030 | .050 | .050 | No |  | 1.00 | 1.00 | 1.00 | 1.00 | No |  | - | - |  | - | - |  | .002 | <.001 | .003 | .002 | No |
| *Access to/availability of services/ destinations* | | | | | |  |  |  |  |  |  |  |  |  |  |  |  |  |  |  |  |  |  |  |  |  |  |  |  |
| Overall access to destinations/services | <.001 | <.001 | <.001 | <.001 | No |  | <.001 | <.001 | <.001 | <.001 | No |  | 1.00 | 1.00 | 1.00 | 1.00 | No |  | 1.00 | 1.00 | 1.00 | 1.00 | No |  | <.001 | <.001 | <.001 | <.001 | No |
| Land use mix – destination diversity | <.001 | <.001 | <.001 | <.001 | No |  | .003 | .003 | .003 | .003 | No |  | .310 | .080 | .380 | .165 | No |  | - | - | - | - | - |  | <.001 | <.001 | <.001 | <.001 | No |
| Shops / commercial | <.001 | <.001 | <.001 | <.001 | No |  | .156 | .156 | .156 | .156 | No |  | .050 | .050 | .050 | .050 | No |  | .050 | .050 | .050 | .050 | No |  | <.001 | <.001 | <.001 | <.001 | No |
| Food outlets | .542 | .404 | .582 | .424 | No |  | .050 | .050 | .049 | .050 | No |  | .050 | .050 | .050 | .050 | No |  | - | - | - | - | - |  | .027 | .021 | .031 | .020 | No |
| Business/ government/ institutional/ industrial | **.112** | **.082** | **.130** | **.033** | **Yes** |  | 1.00 | 1.00 | 1.00 | 1.00 | No |  | .006 | .006 | .006 | .006 | No |  | - | - | - | - | - |  | .018 | .011 | .022 | .009 | No |
| Health and aged-care | .451 | .404 | .469 | .424 | No |  | .166 | .166 | .166 | .166 | No |  | - | - | - | - | - |  | - | - | - | - | - |  | .166 | .176 | .170 | .166 | No |
| Religious | 1.00 | 1.00 | 1.00 | 1.00 | No |  | .166 | .166 | .166 | .166 | No |  | - | - | - | - | - |  | - | - | - | - | - |  | .327 | .327 | .327 | .327 | No |
| Public transport | <.001 | <.001 | <.001 | <.001 | No |  | 1.00 | 1.00 | 1.00 | 1.00 | No |  | 1.00 | 1.00 | 1.00 | 1.00 | No |  | .050 | .050 | .050 | .050 | No |  | <.001 | <.001 | <.001 | <.001 | No |
| Parks / open space / recreation | .001 | <.001 | .002 | <.001 | No |  | .002 | .002 | .002 | .002 | No |  | .287 | .287 | .287 | .327 | No |  | - | - | - | - | - |  | <.001 | <.001 | <.001 | <.001 | No |
| Entertainment | 1.00 | 1.00 | 1.00 | 1.00 | No |  | .191 | .191 | .191 | .191 | No |  | 1.00 | 1.00 | 1.00 | 1.00 | No |  | - | - |  | - | - |  | .553 | .579 | .548 | .565 | No |
| Other | .310 | .310 | .310 | .310 | No |  | - | - | - | - | - |  | 1.00 | 1.00 | 1.00 | 1.00 | No |  | - | - | - | - | - |  | .390 | .417 | .385 | .385 | No |
| *Pedestrian & cycling infrastructure* | | | | | |  |  |  |  |  |  |  |  |  |  |  |  |  |  |  |  |  |  |  |  |  |  |  |  |
| Pedestrian-friendly features | .024 | .023 | .027 | .024 | No |  | .003 | .002 | .003 | .003 | No |  | .194 | .251 | .183 | .129 | No |  | - | - | - | - | - |  | <.001 | <.001 | <.001 | <.001 | No |
| Barriers to walking/cycling | .535 | .601 | .525 | .555 | No |  | .397 | .271 | .424 | .424 | No |  | .295 | .194 | .320 | .258 | No |  | - | - | - | - | - |  | .664 | .491 | .702 | .661 | No |
| Benches / sitting facilities | **.048** | **.111** | **.041** | **.050** | **Yes** |  | .033 | .034 | .032 | .032 | No |  | - | - | - | - | - |  | .674 | .674 | .674 | .674 | No |  | .004 | .010 | .003 | .004 | No |
| Street lights | .290 | .315 | .287 | .327 | No |  | .050 | .050 | .050 | .050 | No |  | .050 | .050 | .050 | .050 | No |  | .595 | .595 | .595 | .595 | No |  | .013 | .044 | .010 | .024 | No |
| Easy access to building entrance | .002 | .002 | .003 | .002 | No |  | .010 | .014 | .009 | .009 | No |  | - | - | - | - | - |  | - | - | - | - | - |  | <.001 | <.001 | <.001 | <.001 | No |
| Public toilets | 1.00 | 1.00 | 1.00 | 1.00 | No |  | 1.00 | 1.00 | 1.00 | 1.00 | No |  | - | - | - | - | - |  | 1.00 | 1.00 | 1.00 | 1.00 | No |  | 1.00 | 1.00 | 1.00 | 1.00 | No |
| *Aesthetics and cleanliness/order* |  |  |  |  |  |  |  |  |  |  |  |  |  |  |  |  |  |  |  |  |  |  |  |  |  |  |  |  |  |
| Greenery and aesthetically pleasing scenery | .189 | .078 | .238 | .115 | No |  | .239 | .202 | .247 | .245 | No |  | 1.00 | 1.00 | 1.00 | 1.00 | No |  | 1.00 | 1.00 | 1.00 | 1.00 | No |  | .160 | .091 | .188 | .108 | No |
| Littering / vandalism / decay | **.050** | **.082** | **.050** | **.059** | **Yes** |  | 1.00 | 1.00 | 1.00 | 1.00 | No |  | .802 | .802 | .802 | .802 | No |  | .883 | .883 | .883 | .883 | No |  | .191 | .233 | .188 | .201 | No |
| Pollution (air, noise) | .293 | .212 | .320 | .258 | No |  | - | - | - | - | - |  | 1.00 | 1.00 | 1.00 | 1.00 | No |  | .963 | .963 | .963 | .963 | No |  | .457 | .373 | .482 | .422 | No |
| *Safety and traffic* |  |  |  |  |  |  |  |  |  |  |  |  |  |  |  |  |  |  |  |  |  |  |  |  |  |  |  |  |  |
| Traffic / pedestrian safety | .484 | .476 | .492 | .547 | No |  | .189 | .231 | .183 | .183 | No |  | **.056** | **.024** | **.069** | **.110** | **Yes** |  | .166 | .166 | .166 | .166 | No |  | .888 | .710 | .931 | .935 | No |
| Human or motorised traffic volume | .024 | .024 | .024 | .024 | No |  | **.054** | **.083** | **.050** | **.050** | **Yes** |  | - | - | - | - | - |  | - | - | - | - | - |  | .004 | .006 | .003 | .003 | No |
| Crime/personal safety | .667 | .953 | .612 | .618 | No |  | 1.00 | 1.00 | 1.00 | 1.00 | No |  | 1.00 | 1.00 | 1.00 | 1.00 | No |  | - | - | - | - | - |  | .755 | .964 | .715 | .697 | No |

*Notes.* p = p value; Diff = difference in conclusions between types of adjustment of article characteristics; subscript ‘a’ = fully adjusted (for sample size and article quality); subscript ‘sa’ = adjusted for sample size; subscript ‘q’ = adjusted for article quality; subscript ‘u’ = unadjusted.

**Comments**

The evidence of positive associations of total walking with benches/sitting facilities and negative associations with littering/vandalism/decay was attenuated when not accounting for article quality. An examination of possible sources of differences in findings indicated that they were both due to two articles with non-validated measures of active travel observing nil associations with either or both environmental attributes and positive associations with littering/vandalism/decay [reference # in review 25, 50].

Slight differences in findings were observed for human or motorised traffic volume (‘crowdedness’), whereby significant positive associations with within-neighbourhood walking for transport were observed only when not accounting for sample size. While weak positive associations were found between ‘crowdedness’ and within-neighbourhood walking for transport in a study on 484 Hong Kong elders [58], no evidence of associations were observed in a more recent study on a larger (909 participants) and more representative sample of the general population of Hong Kong older adults [4].

A significant positive association was found between walking + cycling and traffic/pedestrian safety when adjusting only for sample size. This difference in conclusions was due to a pilot study with a very small sample size reporting a negative effect of traffic/pedestrian safety [47].
